# T cell reactivity to regulatory factor X4 in type 1 narcolepsy

**DOI:** 10.1038/s41598-021-87481-8

**Published:** 2021-04-09

**Authors:** Guo Luo, Selina Yogeshwar, Ling Lin, Emmanuel Jean-Marie Mignot

**Affiliations:** 1 Department of Psychiatry and Behavioral Sciences, Stanford University Center for Sleep Sciences, Stanford University School of Medicine, Palo Alto, CA USA; 2 Division of Biosciences, Department of Cell and Developmental Biology, University College London, Gower Street, London, WC1E 6BT UK

**Keywords:** Autoimmunity, Hypocretin, Sleep, Neuroscience, Circadian rhythms and sleep, Orexin, T cells, Autoimmune diseases, Immunology, Neuroimmunology

## Abstract

Type 1 narcolepsy is strongly (98%) associated with human leukocyte antigen (HLA) class II DQA1*01:02/DQB1*06:02 (DQ0602) and highly associated with T cell receptor (TCR) alpha locus polymorphism as well as other immune regulatory loci. Increased incidence of narcolepsy was detected following the 2009 H1N1 pandemic and linked to Pandemrix vaccination, strongly supporting that narcolepsy is an autoimmune disorder. Although recent results suggest CD4+ T cell reactivity to neuropeptide hypocretin/orexin and cross-reactive flu peptide is involved, identification of other autoantigens has remained elusive. Here we study whether autoimmunity directed against Regulatory Factor X4 (RFX4), a protein co-localized with hypocretin, is involved in some cases of narcolepsy. Studying human serum, we found that autoantibodies against RFX4 were rare. Using RFX4 peptides bound to DQ0602 tetramers, antigen RFX4-86, -95, and -60 specific human CD4+ T cells were detected in 4/10 patients and 2 unaffected siblings, but not in others. Following culture with each cognate peptide, enriched autoreactive TCRαβ clones were isolated by single-cell sorting and TCR sequenced. Homologous clones bearing TRBV4-2 and recognizing RFX4-86 in patients and one twin control of patient were identified. These results suggest the involvement of RFX4 CD4+ T cell autoreactivity in some cases of narcolepsy, but also in healthy donors.

## Introduction

Type 1 Narcolepsy (T1N) is a disabling neurological disorder that affects 1/2000 individuals. It is characterized by excessive daytime sleepiness, disturbed nocturnal sleep, sleep paralysis, hypnagogic hallucinations and sudden episodes of bi-lateral losses of muscle tone, triggered by emotions (cataplexy)^1^. The condition is caused by the loss of (> 90%) orexin/hypocretin (HCRT)-producing neurons^2,3^ and is highly (98%) associated with human leukocyte antigen (HLA) class II haplotype DQA1*01:02/DQB1*06:02 (DQ0602)^4–11^. Furthermore, this disorder is also strongly associated with T cell receptor (TCR) alpha polymorphism and other immune response genes, including cathepsin H (CTSH) and tumor necrosis factor (ligand) superfamily member 4 (TNFSF4)^4,12,13^. Increased incidence of narcolepsy involving hundreds of patients from different European countries including Finland, Sweden, and Norway was detected, following the 2009–2010 influenza A H1N1 pandemic and associated vaccination campaign with Pandemrix^14–24^. It was also reported that cases of T1N were triggered by other environmental factors, including Streptococcus infection, which has previously been associated with a number of autoimmune diseases^15,25–30^. Taken together, this data strongly suggests that T1N is an autoimmune disorder.

So far, no consistent autoantibodies have been found in T1N in spite of large efforts. One of the most promising autoantigens first identified was TRIB2^31^, a protein initially thought to be enriched in HCRT cells. It was shown by three different studies, including one by our group, that TRIB2 autoantibody levels are elevated in recent onset narcolepsy cases^32^. However, later efforts failed to consistently confirm this finding^33,34^.

Similarly, numerous efforts were exerted to screen for autoantibodies against HCRT, HCRT receptor 1 (HCRTR1) and HCRTR2 in T1N patients and healthy controls^35–37^. Although Ahmed et al. report anti-HCRTR2 autoantibodies in the majority of post-Pandemrix narcolepsy patients tested (17 of 20 sera)^38^, others utilizing a similar protocol have reported conflicting results in both sporadic narcolepsy patients (5 of 181, 3 of 61 sera) and post-Pandemrix narcolepsy patients (0 of 40 sera)^37^. Results suggesting association of anti-streptolysin O (ASO) and anti-DNAse B (ADB) antibodies with recent narcolepsy onset could not be replicated either in China^25,27,39^. Notably, Deloumeau et al. identified higher level of IgG complexed with HCRT1 in T1N patients compared to controls, whereby overall serum levels of IgG did not differ between the two groups^40,41^. Recent techniques used have included radioligand-binding^35^, cell-based enzyme-linked immunosorbent (ELISA)^38^ and flow cytometry-based assays^37,42,43^, all with negative results. While the general consensus is now that a T cell mediated response is likely the primary mechanism underlying T1N, it yet remains an enigma whether this cascade may also generate a humoral response with the presence of any antibodies specifically and reliably associated with T1N remaining contentious.

In contrast to the findings concerning antibody-mediated immunity, adaptive cell-mediated immunity has been increasingly and robustly implicated in the pathophysiology of narcolepsy^44,45^, a result that fits well with genetic data indicating association with HLA and TCR loci. Thanks to HLA class I and II multimers and fluorescence-activated cell sorting (FACS)-based single cell sorting and sequencing technologies, investigation of cellular immune responses to infectious agents and autoantigens in autoimmune diseases is now possible in more details^46,47^. Specifically, tetramer HLA-targeted T cells facilitates identification of antigen specific TCR-groups within highly diverse TCR repertoires^48,49^, which are stochastically generated by V(D)J recombination, a process rearranging germline TCR loci in each T cell^50,51^. Using this technology, there is increasing evidence that CD4 autoreactivity directed against HCRT itself may indeed be a major autoreactive process in human narcolepsy^46,52^. Of note, it was previously shown in a mouse model of narcolepsy that both pathogenic CD4 Th1 cells, as well as cytotoxic CD8 T cells readily infiltrate the hypothalamus, whereby the latter cell-type also lead to a specific destruction of hypocretin neurons and a narcolepsy-phenotype in these animals^53^.

In this study, we hypothesize that regulatory factor X4 (RFX4) might be another important CD4+ T cell autoantigen in T1N, due to its high enrichment in HCRT cells^34,54,55^. RFX members share a highly-conserved deoxyribonucleic acid (DNA)-binding domain^56^ and regulate expression of their target genes, including major histocompatibility complex class II (MHCII) and interleukin-5 receptor α chain (IL-5Rα)^57–64^. Six isoforms of RFX4 have been identified^65^. While RFX4 variant 1 (RFX4_v1), RFX4_v2 and RFX4_v4 are specifically expressed in testis^62,66^, expressions of both RFX4_v5 and RFX4_v6 is observed exclusively in gliomas^66^. RFX4_v3 is specifically expressed in the brain and is crucial for brain morphogenesis, being involved in many aspects of brain development and disease^65–70^. Of notable interest is the fact RFX4_v3 is expressed in the suprachiasmatic nucleus (SCN), the mammalian pacemaker responsible for generating a 24-h-circadian rhythm^71–73^. It can also be induced by light exposure in a subjective night-specific manner, a typical characteristic of several other core SCN circadian genes^74^, suggesting a role in circadian regulation. This hypothesis is further supported by findings of Glaser and colleagues (2005), who identified RFX4_v3 as a potential risk factor for Bipolar disorder (BPD)^75^, a condition frequently featuring severe circadian instability^76–78^. Intriguingly, RFX4 is highly enriched in-, and significantly specific to HCRT neurons, according to the results of translating ribosome affinity purification (TRAP)^34,54,55^. Nevertheless, little is known about the regulatory network of RFX4 and the role it exerts on HCRT neurons. Taken together, the implication of RFX4 in circadian regulation, its hypocretin neuron specific expression and the effect of related members on MHC class II genes, renders this protein an interesting candidate for investigation as an autoantigen in T1N. Of additional interest is the fact CD8+ directed T cell activity against this intracellular antigen has also been shown in a recent study of narcolepsy patients^45^.

To further explore the hypothesis that immune reactivity directed toward RFX4 is relevant to narcolepsy pathophysiology and hypocretin cell loss, we searched for autoantibodies directed against RFX4_v1/v3/v4 in 86 T1N patients and 88 controls using high throughput flow cytometry-based (FACS) staining assays and explored T cell reactivity to RFX4 peptides bound to DQ0602. FACS positive samples for autoantibodies were confirmed using cell-based immunostaining and western blotting. While no autoantibodies were found, T cell binding DQ0602-RFX4 tetramers were found in four patients and two sibling controls of cases. The corresponding RFX4 antigen, DQ0602 restricted specific CD4+ T cells were next single-cell sorted for TCR sequencing and unique clone enrichment and preferential v-gene segments in TCR repertoires observed. Finally, activation of enriched TCR clones by RFX4 peptides was studied in vitro, showing functional effects. Altogether, our results provide evidence for the involvement of RFX4 in some T1N cases and also healthy donors.

## Materials and methods

### Ethics statement

This study has been reviewed and approved by the Stanford University Institutional Review Board (Protocol # 14325, Registration # 5136). Informed consent was obtained from each participant. We confirm that all methods were carried out in accordance with relevant guidelines and regulations.

### Participants

For anti-RFX4 autoantibody detection, two groups of matched T1N patients and healthy controls were created. One group consists of 39 post-Pandemrix T1N (PP-N) cases (mean age ± standard error of the mean (SEM): 14.9 ± 1.26, 30% male) and 18 post-Pandemrix controls (PP-C) (mean age ± SEM: 13.17 ± 0.97, 39% male), the other group consists of 47 recent early onset T1N (EO-N) cases (mean age ± SEM: 12.95 ± 1.43, 58% male) and 70 other controls (O-C) (mean age ± SEM: 18.85 ± 1.31, 46% male) (Table 1, Dataset S1). All patients meet international classification of sleep disorders 3 (ICSD3) criteria (http://www.aasmnet.org/store/product.aspx?pid=849) for T1N. All are DQ0602 positive with one exception, a patient known to have low cerebrospinal fluid (CSF) hypocretin-1 levels. For T cell reactivity examination, 2 early onset T1N patients, 7 post-Pandemrix T1N patients, 5 post-Pandemrix controls, and 1 other control were tested and detailed information was summarized in Table 1 and Dataset S2. All 15 subjects are DQ0602 positive. Flow cytometry dot plots of 10 out of 15 subjects were also shown in S11 in Luo et al.^46^.

**Table 1:** Summary of subjects.

	Autoantibody screening			Tetramer staining		
	No	Percentage	Age ± sem	No	Percentage	Age ± sem
**Female**						
Patient	46	26.4	14.4 ± 1.3	4	26.7	20.1 ± 2.6
Control	49	28.2	16.6 ± 0.7	4	26.7	27.1 ± 7.8
Subtotal	95	54.6	15.5 ± 0.7	8	53.3	23.6 ± 4.0
**Male**						
Patient	40	23.0	13.7 ± 1.5	5	33.3	17.9 ± 2.5
Control	39	22.4	19.1 ± 1.5	2	13.3	27.5 ± 6.0
Subtotal	79	45.4	16.3 ± 1.1	7	46.7	20.6 ± 2.8
Total	174			15		

*Sem* standard error of the mean.

### Vaccine

Pandemrix (A/California/7/2009 (H1N1) NYMC X-179A monovalent bulk (inactivated, sterile)) (Batch# AFLSFDA280) was used throughout the study. It was manufactured with HA content at 139 μg/ml (determined with single radial diffusion (SRD)) by GlaxoSmithKline (GSK) Dresden in January 2010. This batch has been used during the 2009–2010 vaccination campaign in Europe. Summary of Pandemrix characteristics can be found in the European Medicines Agency library (http://www.ema.europa.eu/docs/en_GB/document_library/Other/2010/05/WC500091295.pdf).

### RFX4 constructs

Original clones of RFX4_v1 (Cat# RG205481, OriGene), RFX4_v3 (Cat# RG223334, OriGene), RFX4_v4 (Cat# RG233113, OriGene) and green fluorescent protein (GFP) expression vector pCMV6-AC-GFP (Cat# PS100010, OriGene) were purchased from OriGene. These were transformed to TOP10 competent cells (Cat# C404003, Invitrogen) and plasmids purified from bacterial culture using EndoFree plasmid maxi kit (Cat# 12362, QIAGEN).

### Anti-RFX4 autoantibody detection with flow cytometry

Detailed protocol and quantitation analysis methods have been described previously^42,43^. Briefly, human embryonic kidney (HEK) 293 T cells (ATCC CRL-3216) were cultured in Dulbecco’s modified Eagle’s medium (DMEM) (Cat# ATCC 30-2002, ATCC), supplemented with 10% fetal bovine serum (FBS) (Cat# 26140079, Gibco) and 1% penicillin–streptomycin (Pen/Strep) (Cat# 15140-122, Gibco) in a 75cm^2^ tissue culture flask (REF# 658175, CELLSTAR) at 37 °C, 5% CO_2_, and transfected with RFX4_v1, RFX4_v3, RFX4_v4 or pCMV6-AC-GFP using the Lipofectamine 3000 reagent (Cat# L3000015, Invitrogen) in Opti-MEM I reduced serum medium (REF# 31985070, Gibco). After 24 h, cells were harvested and stained with Zombie NIR (Cat# 423106, BioLegend), followed by fixation and permeabilization with fix/perm buffer set (Cat# 421403, BioLegend) according to the manufacturer’s instructions. Validation and sensitivity were detected using two positive antibodies: mouse anti-RFX4 antibody (Cat# H00005992-B01P, Abnova) (Supplemental Fig. S1) and rabbit anti-RFX4 antibody (Cat# NBP2-48967, Novus) (Supplemental Fig. S2), followed by staining with R-phycoerythrin (PE) anti-mouse immunoglobulin (Ig)G (REF# 12-4010-82, eBioscience) and PE anti-rabbit IgG (REF# 12-4739-81, eBioscience) (1:100), respectively. Cells were incubated with human serum (1:20) for 1 h at 4 °C, followed by staining with PE anti-human Igλ antibody (REF# 12-9990-42, eBioscience) (1:100) and PE anti-human Igκ antibody (REF# 12-9970-42, eBioscience) (1:100). Nonspecific binding was removed by washing with 0.05% Tween-20 in PBS. Cells were run on BD LSRII and data were analyzed with FlowJo^79^.

Quantitation analysis methods were modified as follows: for each sample, mean fluorescence intensities (MFI) of PE channel (MFI^PE^) within live GFP positive HEK293T cells (HEK293T^GFP+^) and GFP negative HEK293T cells (HEK293T^GFP−^) were calculated, then ΔMFI^PE^ was determined by subtracting MFI^PE^ of HEK293T^GFP-^ from MFI^PE^ of HEK293T^GFP+^. Cut-off value of ΔMFI^PE^ was determined as the mean of ΔMFI^PE^ + 3 × standard deviation (SD) of control samples^35^.

### Cell-based assay (CBA) for autoantibody detection under microscope

A protocol described previously^36^ was used. Briefly, HEK293T cells were cultured and transfected (as described above) on glass coverslip (REF# 354085, Corning) in a 12-well plate (REF# 353043, Corning). After 24 h, cells were fixed and permeabilized, and then incubated with human serum (1:20) or rabbit anti-RFX4 antibody (1:500) for 2 h at room temperature (RT) in the dark, followed by Alexa Fluor (AF) 555-conjugated anti-human IgG antibody (Cat# A-21433, ThermoFisher) (1:500) or AF555-conjugated anti-rabbit IgG antibody (Cat# A-21428, ThermoFisher) (1:500), respectively. All antibodies or sera were diluted in perm buffer and cells were washed twice with perm buffer after each incubation. Images were taken by EVOS FL imaging system (Cat# AMF4300, ThermoFisher). Images were analyzed using ImageJ2.0.0 image processing software^80^. An area in the region of interest was selected and all DAPI + GFP + and DAPI + GFP + AF555 + cells were counted using the cell counter plugin in ImageJ2.0.0^80^.

### Immunoprecipitation (IP)

HEK293T cells (equal aliquot) were cultured and transfected in 75 cm^2^ flasks as described above. After 24 h, cells were harvested, washed with cold phosphate buffered saline (PBS) (Cat# 10010-049, Gibco) and lysed in RIPA buffer (Cat# 89900, ThermoFisher) supplemented with complete protease inhibitor (REF# 11873580001, Roche) on ice for 15 min. Debris was removed by centrifugation at 12,000 rpm for 15 min. Supernatant was incubated with anti-tGFP-conjugated agarose beads (Cat# TA183081, OriGene) overnight at 4 °C by end-to-end rotation. These beads were washed 3 times and boiled at 95 °C for 10 min after adding 50 μl of 4× Laemmli buffer (Cat# 1610747, Bio-Rad). Supernatant was loaded to a precast gel (Cat# 4561093, Bio-Rad) for western blotting.

### Western blotting

For western blotting protocol, see https://www.bio-rad.com/webroot/web/pdf/lsr/literature/Bulletin_2895.pdf. Briefly, proteins were transferred from gel to a PVDF membrane (Cat# IPVH00010, EMD Millipore) and then incubated with primary anti-RFX4 antibody (Cat# H00005992-B01P, Abnova) (1:2000) or human serum (1:50) (200 μl in 10 ml of blocking buffer) for 2 h at RT, followed by secondary peroxidase-conjugated anti-mouse IgG antibody (Code# 715-035-150, Jackson ImmnuoResearch) (1:5000) or anti-human Igλ (NBP1-73715, Novus) (1:5000) and anti-human Igκ (NB7466, Novus) (1:5000), respectively. Non-specific binding was removed through extensive washing of 3 × 10 min with 0.05% Tween-20 in PBS. Chemiluminescent substrate (Prod# 32106, ThermoFisher) was applied to the blot and film (Cat# 28906838, GE Healthcare) was developed with a tablet processor (Item# SRX-101A, Konica).

### RFX4 peptide library

An overlapping 15-mer peptide (11-amino acid overlap) library covered 6 RFX4 variants. Each peptide was synthesized with > 90% purity at GenScript and dissolved in dimethyl sulfoxide (DMSO) at a stock concentration of 5 mM.

### Competition binding assay

Peptide binding assay was conducted as previously described^81,82^. Briefly, DQ0602 was incubated with biotinylated EBV epitope (Bio-EBV_486–500_, Bio-(GGG)RALLARSHVERTTDE) and RFX4 peptide for 72 h at 37 °C, followed by incubation with monoclonal anti-DQ (SPV-L3) antibody (Cat# BNUM0200-50, Biotium) in a high binding 96-well plate (REF# 9018, Corning). DELFIA time-resolved fluorescence (TRF) intensity was detected using a Tecan Infinite M1000 after successive incubation with Europium (Eu)-labelled streptavidin (Cat# 1244-360, PerkinElmer) and enhancement solution (Cat# 1244-105, 9 PerkinElmer) (Supplemental Fig. S7A). Plate was washed 5 times with 300 µl/well wash buffer (0.05% Tween-20 in PBS) to remove nonspecific binding. Bio-EBV486-500 alone is as the reference (positive control) and without any peptide is as negative control. Compared with the reference, RFX4 peptides with lower than 25% and 25–50% of fluorescence are considered as strong and weak peptides, respectively.

### Tetramer DQ0602-peptide staining and single cell TCR sequencing (TCRseq)

The full protocol was described previously^81,83^. In brief, biotinylated DQ0602 was loaded with peptide at pH 5.2 and incubated with PE-conjugated streptavidin. Peripheral blood mononuclear cells (PBMCs) were stained with concentrated and purified tetramer DQ0602-peptide and antibody cocktail, followed by Fluorescence-activated cell sorting (FACS) (Supplemental Figs. S4, S6, S7). Tetramer DQ0602-peptide positive CD4+ T cells were single-cell sorted into 96-well plates for single cell TCR sequencing.

### Luciferase activity

TCRαβ chains tagged with GFP were cloned to N103 vector (a gift from Dr. Mark Davis) by IDT (Integrated DNA Technologies, Inc.). Jurkat 76 (J76)-NFATRE-luc cells (a gift from Dr. Mark Davis) were transfected with TCR using the Lipofectamine 3000 reagent (Cat# L3000015, Invitrogen). TCR positive cells were sorted using anti-human TCRα/β antibody (Cat# 306702, BioLegend). In 200 µl of complete RPMI medium (RPMI (Cat# 61870-036, Gibco) supplemented with 10% FBS and 1% Pen/Strep), 0.1 million of J76-TCR cells were co-cultured with 0.1 million of RM3-DQ0602^84^ cells at the presence of 10 µM of pHA_273–287_, NP_17–31_, HCRT_54–66–NH2_, HCRT_86–97–NH2_, or RFX4-43, or 5 µM of RFX4-86, RFX4-95, or RFX4-60 or 0.2 µl DMSO for 8 h. Luciferase activity was detected with Nano-Glo Luciferase Assay Kit (Cat# N1130, Promega).

### Statistical analysis

For anti-RFX4 autoantibody analysis and luminescence of J76-TCR, values were expressed as mean ± SD and mean ± Standard Error of the Mean (SEM), respectively. Statistical comparisons were calculated with two-tailed *t*-tests. Data were plotted using GraphPad Prism version 5.0.0 for Windows (GraphPad Software, San Diego, California USA, www.graphpad.com) and R^85^. p-value < 0.05 was considered statistically significant.

## Results

### Autoantibodies against RFX4 are absent or very rare in cases and controls

**Figure 1:**
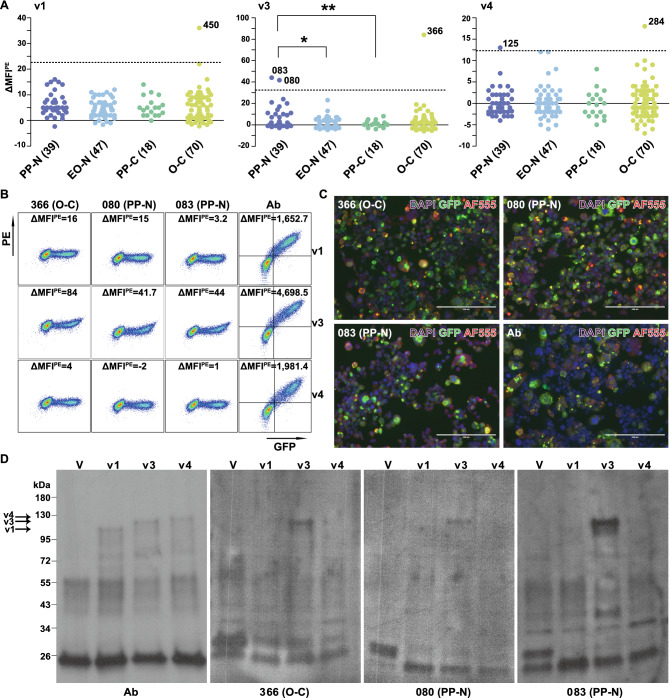
Anti-RFX4 autoantibody detection. (**A**) Sera were screened using HEK293T cells expressing GFP tagged RFX4 variants and PE-conjugated anti-human Igλ/κ antibody. Analysis was performed between groups of post-Pandemrix T1N (PP-N) patients, post-Pandemrix controls (PP-C), recent early onset T1N (EO-N) patients, and other controls (O-C) for RFX4_v1, RFX4_v3, and RFX4_v4. Each dot corresponds to one subject. ΔMFI^PE^ = (MFI^PE^ of HEK293T^GFP+^) − (MFI^PE^ of HEK293T^GFP-^). Cut-off value of ΔMFI^PE^, which is denoted by dotted line, was determined as the mean of ΔMFI^PE^ + 3 × standard deviation (SD) of control samples. Subject IDs above cut-off value and statistical significance are shown. *p < 0.05; **p < 0.01. It was independently repeated once. Data were plotted using GraphPad Prism version 5.0.0 for Windows (GraphPad Software, San Diego, California USA, www.graphpad.com). (**B**) FACS dot plots of three subjects in anti-RFX4 autoantibody detection. HEK293T cells expressing RFX4_v1, v3 and v4-GFP were incubated with human serum (1:20) or positive rabbit anti-RFX4 antibody, followed by PE anti-human Igλ/κ antibody (1:100) or PE anti-rabbit IgG antibody (1:100), respectively. Representative dot plots of 3 subjects and positive antibody (1:500) with % of each quadrant population in live single cells are shown. Ab, antibody. It was independently repeated once. Plots were analyzed with FlowJo (version 10.0.8, Becton, Dickinson and Company). (**C**) Anti-RFX4_v3 autoantibody detection using cell-based immunostaining and observation under microscope. HEK293T cells expressing GFP-tagged RFX4_v1, v3, and v4 were stained with human serum (1:20) or rabbit anti-RFX4 antibody (Ab) (1:500), followed by AF555-conjugated anti-human IgG antibody (1:500) or AF555-conjugated anti-rabbit IgG antibody (1:500), respectively. 3 subjects with ΔMFI^PE^ above cut-off value of RFX4_v3 were tested. Bar = 200 μm. It was performed once. Images were analyzed using ImageJ2.0.0 (NIH). (**D**) Anti-RFX4 autoantibody detection using western blotting. HEK293T cells expressing GFP-tagged RFX4_v1, v3, v4 and vector were lysed and anti-tGFP-conjugated agarose beads were incubated with supernatant for Co-IP. Positive antibody and three subjects with ΔMFI^PE^ above cut-off value were tested. RFX4_v1, RFX4_v3 and RFX4_v4 are indicated in positive antibody staining. V, vector. Cell lysates were loaded into two 10-lane gels and proteins were transferred to two polyvinylidene difluoride (PVDF) membranes. Each membrane was cut into two pieces for incubation with different sera and secondary antibodies. All four pieces of membranes were developed in one cassette for the same exposure. Different contrast was applied to each piece after cropping with Illustrator (Version 16.0.0, Adobe). The full-length gel is included in the supplementary files (Supplemental Fig. S5). It was performed once.

To test for the presence of autoantibodies directed against RFX4 in T1N cases, RFX4_v1, RFX4_v3 and RFX4_v4 tagged with green fluorescent protein (GFP) were expressed in human embryonic kidney (HEK) 293 T cells. Mean fluorescence intensities (MFI) of R-phycoerythrin (PE) were recorded using quantitative flow cytometry-based assay after staining HEK293T cells with sera of 39 post-Pandemrix T1N (PP-N) cases, 18 post-Pandemrix controls (PP-C), 47 recent early onset T1N (EO-N) cases, and 70 other controls (O-C), and secondary PE-conjugated anti-human immunoglobulin (Ig). Comparing ΔMFI^PE^ determined by subtracting MFI^PE^ of HEK293T^GFP-^ from MFIPE of HEK293T^GFP+^, no significant difference was observed between patients and controls for either v1, v3, or v4, however, anti-RFX4_v3 autoantibody levels in PP-N cases were significantly higher compared to patients without vaccination and vaccinated controls (p-value = 0.018 and 0.006, respectively) (Fig. 1A). Out of 174 subjects, autoantibody levels of anti-RFX4_v1, v3 and v4 above cut-off were detected in one (O-C), three (2 PP-N and 1 O-C) and two (1 PP-N and 1 O-C) subjects, respectively (Fig. 1A, Dataset S3), thus suggesting anti-RFX4 autoantibodies are rare. Furthermore, two PP-N patients and one O-C showed positive correlations between PE and GFP signals for RFX4_v3, but not for v1 or v4 (Fig. 1B), which was confirmed by cell-based immunostaining assay (CBA) (Fig. 1C) and using western blotting (Fig. 1D), therefore validating results attained using high sensitive flow cytometry-based assay. Taken together, these findings show that autoantibodies against RFX4 are very rare, and are unlikely to mediate pathology in T1N, as already suggested by previous studies^35–37^.

### Detection of RFX4 antigen specific CD4+ T cells

**Figure 2:**
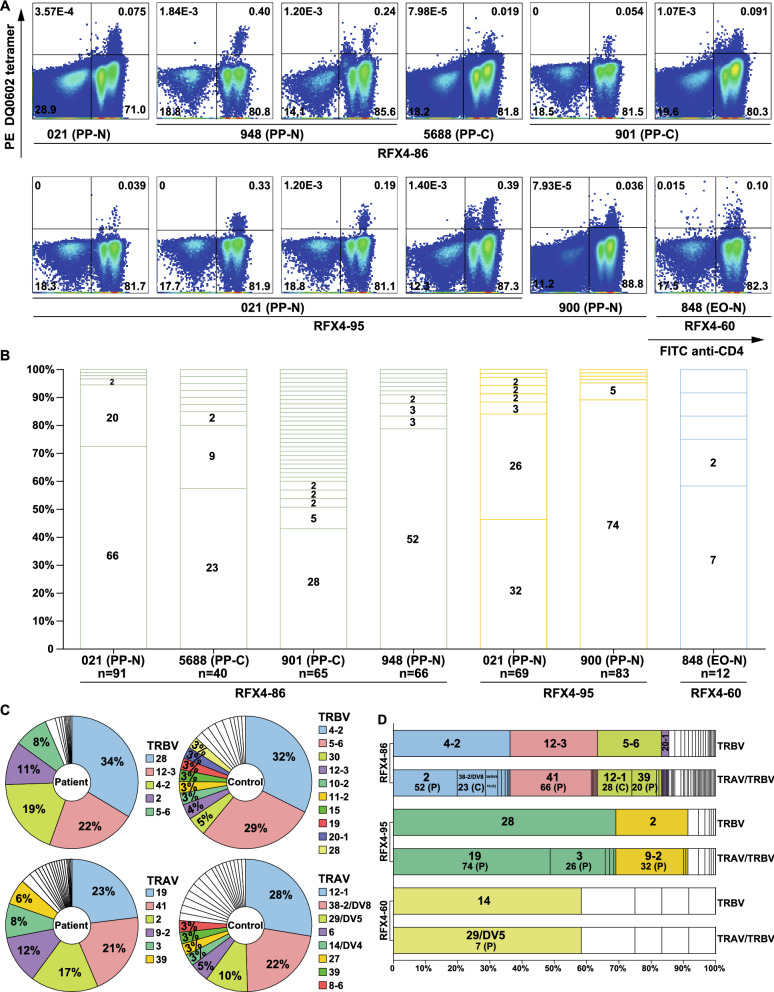
RFX4 antigen specific CD4+ T cell receptor repertoire. (**A**) FACS dot plots of tetramer DQ0602-RFX4 staining, PBMCs were stimulated with 6.25 μM RFX4 peptide in a 96-well plate (1–2.5 × 10^6^ cells/ml) for 10 days at 37 °C, 5% CO_2_. 20 IU/ml IL2 was supplemented from day 8 to day 10. Cultured cells were incubated with PE-labelled tetramer DQ0602-RFX4 peptide for 90 min at 37 °C, 5% CO_2_, followed by staining with BV421 anti-CD3, FITC anti-CD4 and AF700 anti-CD8 antibodies on ice. Live single CD3 + T cells with percentage of each quadrant population are shown. *EO-N* early onset patient, *PP-N* post-Pandemrix patient, *PP-C* post-Pandemrix control,*O-C* other controls. It was independently repeated at least once. Plots were analyzed with FlowJo (version 10.0.8, Becton, Dickinson and Company). (**B**) Paired TCR enrichment. Tetramer DQ0602 positive CD4+ CD8− T cells were single-cell sorted directly into 96-well plates, followed by 3 nested PCR reactions and TCR sequencing pipeline. TCRαβ counts in each subject-peptide reaction. Each slice represents a unique TCRαβ clone. The number of single cells for each unique clone is shown in the slices. Unnumbered slices represent a unique clone that was observed only once. The total number of single cells (n) for each subject-peptide reaction is displayed on x-axis below subject. Graph was created with Illustrator (Version 16.0.0, Adobe). (**C**) Usage of V-gene segments in patient and control paired TCR repertoire. Single cells obtained from all patients or controls respectively, using the same TRAV or TRBV gene, were grouped into one category. Each slice represents a unique TRAV or TRBV gene (see adjacent legend) and the percentage of cells using it. TRAVs and TRBVs used by less than 3% of cells are represented by slices without color and number. Graph was created with Illustrator (Version 16.0.0, Adobe). (**D**) TRBV and corresponding TRAV/TRBV pairings to each RFX4-86/95/60 peptide. Each slice represents a unique TRBV gene or TRAV/TRBV pairing. Slices show TRBV or TRAV genes used by more than 5 single cells and the same TRBV gene is represented by the same color. In the TRAV/TRBV pairing bar, the top digit describes the particular TRAV gene and the bottom number the total number of single cells using each respective gene (and whether they were isolated from patients P or controls C). Diverse TRBV and TRAV/TRBV pairings expressed by fewer than 5 single cells are represented by bars without color and annotation. TCR sequencing was performed once. Graph was created with Illustrator (Version 16.0.0, Adobe).

To detect antigen-specific CD4+ T cells, peripheral blood mononuclear cells (PBMCs) of 3 EO-N, 7 PP-N, 5 PP-C and 1 O-C were cultured with Pandemrix or a single RFX4 peptide and then stained with tetramer DQ0602, loaded by 32 strong and 6 weak RFX4 peptides (Table 2). Sporadic pattern of Pandemrix (Supplemental Fig. S3) stimulation suggested no cross reactivity between Pandemrix and RFX4. Notably, stimulation with the three different peptides, RFX4-60, -86 and -95, which are strong peptides and shared in at least four RFX4 variants, resulted in clearly separated populations of specific CD4+ T cells in four patients and two sibling controls of cases (Fig. 2A), suggesting T cell response engagement with RFX4 in these narcoleptic cases and controls.

**Table 2:** RFX4 peptides for tetramer DQ0602.

Peptide	Position	Sequence	Specific or Shared	Affinity	% EBV
RFX4-2	v1 5–19	AFGGSEFFIPEGIQI	v1	SB	12.66
RFX4-3	v1 9–23	SEFFIPEGIQIDSRC	v1	SB	16.8
RFX4-9	v1 33–47	YHYYGIAVKESSQYY	v1, v2, v3, v4	SB	22.65
RFX4-22	v1 85–99	PEFPNVKDLNLPASL	v1, v2, v3, v4	SB	23.39
RFX4-23	v1 89–103	NVKDLNLPASLPEEK	v1, v2, v3, v4	SB	15.16
RFX4-25	v1 97–111	ASLPEEKVSTFIMMY	v1, v2, v3, v4	SB	8.54
RFX4-26	v1 101–115	EEKVSTFIMMYRTHC	v1, v2, v3, v4	SB	18.89
RFX4-29	v1 113–127	THCQRILDTVIRANF	v1, v2, v3, v4	SB	14.62
RFX4-30	v1 117–131	RILDTVIRANFDEVQ	v1, v2, v3, v4	SB	10.27
RFX4-36	v1 141–155	MPPHMLPVLGSSTVV	v1, v2, v3, v4	WB	25.86
RFX4-41	v1 161–175	CDSILYKAISGVLMP	v1, v2, v3, v4	SB	22.74
RFX4-42	v1 165–179	LYKAISGVLMPTVLQ	v1, v2, v3, v4	SB	10.33
RFX4-43	v1 169–183	ISGVLMPTVLQALPD	v1, v2, v3, v4	SB	10.37
RFX4-60	v1 237–251	QASRTVIHSADITFQ	v1, v2, v3, v4	SB	20.08
RFX4-61	v1 241–255	TVIHSADITFQMLED	v1, v2, v3, v4	WB	25.27
RFX4-72	v1 285–299	QLYQEFDHLLEEQSP	v1, v2, v3, v4, v5, v6	SB	17.49
RFX4-73	v1 289–303	EFDHLLEEQSPIESY	v1, v2, v3, v4, v5, v6	SB	23.54
RFX4-76	v1 301–315	ESYIEWLDTMVDRCV	v1, v2, v3, v4, v5, v6	SB	12.95
RFX4-82	v1 325–339	SLKKVAQQFLLMWSC	v1, v2, v3, v4, v5, v6	SB	23.25
RFX4-86	v1 341–355	GTRVIRDMTLHSAPS	v1, v2, v3, v4, v5, v6	SB	7.05
RFX4-87	v1 345–359	IRDMTLHSAPSFGSF	v1, v2, v3, v4, v5, v6	SB	16.9
RFX4-89	v1 353–367	APSFGSFHLIHLMFD	v1, v2, v3, v4, v5, v6	SB	19.61
RFX4-95	v1 377–391	LHCQERANELMRAMK	v1, v2, v3, v4, v5, v6	SB	12.26
RFX4-96	v1 381–395	ERANELMRAMKGEGS	v1, v2, v3, v4, v5, v6	SB	17.04
RFX4-98	v1 389–403	AMKGEGSTAEVREEI	v1, v2, v3, v4, v5, v6	WB	25.87
RFX4-100	v1 397–411	AEVREEIILTEAAAP	v1, v2, v3, v4, v5, v6	SB	14.93
RFX4-101	v1 401–415	EEIILTEAAAPTPSP	v1, v2, v3, v4, v5, v6	SB	19.99
RFX4-105	v1 417–431	PSFSPAKSATSVEVP	v1, v2, v3, v4, v5, v6	WB	25.99
RFX4-112	v1 445–459	TGLSTTGAMQSYTWS	v1, v3	WB	26.43
RFX4-115	v1 457–471	TWSLTYTVTTAAGSP	v1, v3, v4, v5, v6	SB	17.75
RFX4-158	v1 629–641	AYINGEASTGWAK	v1, v3, v4, v5, v6	SB	20.21
RFX4-171	v3 53–67	SKPHSTPATLQWLEE	v2, v3, v4	SB	8.41
RFX4-172	v3 57–71	STPATLQWLEENYEI	v2, v3, v4	SB	18.78
RFX4-173	v3 61–75	TLQWLEENYEIAEGV	v2, v3, v4	WB	25.64
RFX4-175	v3 69–83	YEIAEGVCIPRSALY	v2, v3, v4	SB	23.99
RFX4-189	v3 125–139	SKYHYYGIAVKESSQ	v2, v3, v4	SB	16.99
RFX4-190	v3 129–143	YYGIAVKESSQYYDV	v1, v2, v3, v4	SB	22.69
RFX4-202	v4 548–562	TGLSTTGAMQAYTWS	v4, v5, v6	SB	9.32

*SB* strong binder, *WB* weak binder.

T cell reactivity to RFX4-86 presented by DQ0602 was detected in both 2 PP-N (021 and 948) and 2 PP-C subjects (5688 and 901) while recognition of RFX4-95 was only found in 2 PP-N patients (021 and 900) (Fig. 2A). T cell reactivity to RFX4-60 was solely detected in one EO-N patient (848) with a tail-like pattern (Fig. 2A). Notably, patient 021 reacted to both RFX4-86 and RFX4-96, which are shared in all 6 RFX4-variants (Table 2). Altogether, these findings reveal RFX4-specific CD4+ T cells in both post-Pandemrix and early-onset narcolepsy patients, therefore suggesting involvement of RFX4 in T cell immunity of theses narcoleptic cases and controls.

### RFX4 antigen specific single CD4+ T cell TCR sequencing

In the next step, our aim was to determine the identity of RFX4-specific CD4+ T cells. Antigen specific CD4+ CD8- T cells of 7 positive reactions to the three peptides RFX4-86, -95, and -60 in 6 subjects were single-cell sorted into 96-well plates for TCRα and β sequencing (i.e. 7 × 96 cells in total) (Dataset S4). For each peptide-subject reaction, more than 65 out of 96 (68%) cells were recovered with pairing TCRαβ, except for RFX4-86/5688 (twin control of case, 40 cells, 42%) and RFX4-60/848 (PP-N, 12 cells, 13%) (Fig. 2B, Dataset S4). At least one unique TCR clone was enriched over 43% (≥ 7 counts). For example, two TCRs were enriched in one patient (021) recognizing either RFX4-86 (73%, 22%) or RFX4-95 (46%, 38%), while one TCR clone with different sequences was enriched in one patient (948, 79%) and two controls (5688, 58%; 901, 43%) recognizing RFX4-86, one patient (900, 89%) recognizing RFX4-95, and one patient (848, 58%) recognizing RFX4-60 (Fig. 2B). Notably, RFX4-86 TRBV4-2, which is associated with T1N^13^, was also enriched in one patient (79%) and one control (58%) (Fig. 2B).

Next, the usage of TCR V gene segments in patients and controls was analyzed. TRBV28 (34%) and TRBV4-2 (32%) were the two most frequently used V gene segments in patients and controls respectively (Fig. 2C). Notably, TRBV4-2, which has previously been associated with T1N^13^, was also frequently used in patients (19%) and controls (32%). Contrastingly, however, frequently used TRAV genes showed significantly more diversification between patients and controls (Fig. 2C).

Frequent usage of TRBV and TRAV/TRBV pairings was distinct between RFX4-86, -95, and -60 (Fig. 2C,D). In RFX4-86, TRBV4-2 (36%) most frequently paired to TRAV2 (52 counts, 20%) in patients and TRAV38-2/DV-8 (23 counts, 9%) in controls, while for RFX4-60, TRBV14 (58%) paired to TRAV29/DV5 (7 counts, 58%) in patients (in controls, no pairing of more than 5 cells could be identified). In RFX4-95, TRBV28 (69%) paired to TRAV19 (74 counts, 49%) and TRAV3 (26 counts, 17%) in patients (Fig. 2D). Taken together, these findings show that TCR is enriched in tetramer DQ0602 positive CD4+ T cells and V gene segments are partially similar between patients and controls, however, differ significantly between RFX4-86, -95 and -60.

### TRBV4-2 in RFX4 autoreactive CD4+ T cells

**Figure 3:**
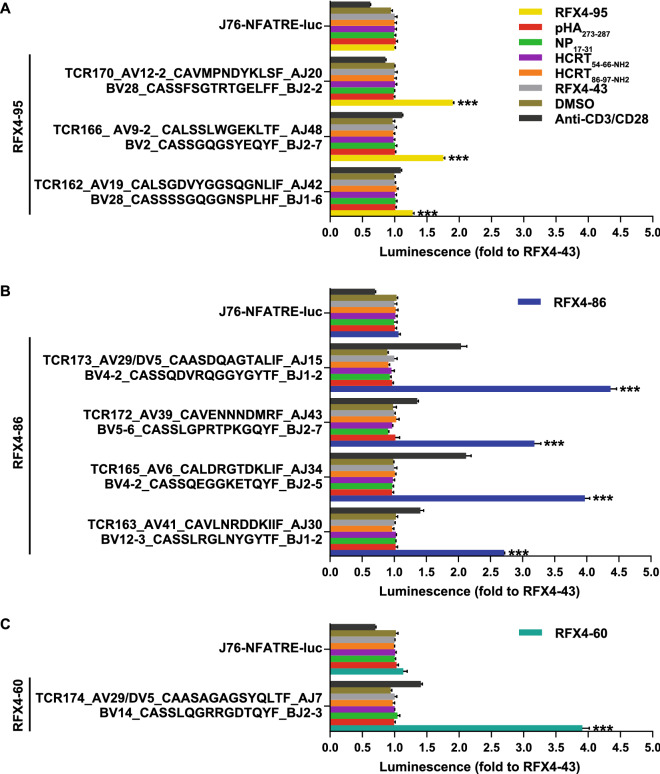
Activation of TCR signaling by RFX4 antigens presented by DQ0602. Jurkat 76-NFATRE-luc cells were transfected with TCRαβ and then co-cultured with RM3-DQ0602 presented with 10 µM of pHA_273–287_, NP_17–31_, HCRT_54–66–NH2_, HCRT_86–97–NH2_, or RFX4-43, or 5 µM of RFX4-86, RFX4-95, or RFX4-60, or 0.2 µl DMSO for 8 h. Luciferase activity was detected in a plate (n = 3). Luminescence fold to RFX4-43 was shown. ***p < 0.001; **p < 0.01; *p < 0.05. It was performed at least once. Graph was created with Illustrator (Version 16.0.0, Adobe).

In a last step, our aim was to probe the reactivity of specific TCRs to RFX4-86, RFX4-95 and RFX4-60. Therefore, enriched TCRαβs (≥ 7 clones, Table 3) were selected and expressed in Jurkat 76 (J76)-NFATRE-luc cells, which lack native TCRs and subsequently co-cultured with antigen presenting cells (APCs) RM3-DQ0602. Luciferase activity showed that TCR162 (TRBV28_CASSSSGQGGNSPLHF_TRBJ1-6/TRAV19_CALSGDVYGGSQGNLIF_TRAJ42), TCR166 (TRBV2_CASSGQGSYEQYF_TRBJ2-7/TRAV9-2_CALSSLWGEKLTF_TRAJ48) and TCR170 (TRBV28_CASSFSGTRTGELFF_TRBJ2-2/TRAV12-2_CAVMPNDYKLSF_TRAJ20) significantly reacted to RFX4-95. Four TCRs showed reactivity against RFX4-86, namely TCR163 (TRBV12-3_CASSLRGLNYGYTF_TRBJ1-2/TRAV41_CAVLNRDDKIIF_TRAJ30), TCR165 (TRBV4-2_CASSQEGGKETQYF_TRBJ2-5/TRAV6_CALDRGTDKLIF_TRAJ34), TCR172 (TRBV5-6_CASSLGPRTPKGQYF_TRBJ2-7/TRAV39_CAVENNNDMRF_TRAJ43) and TCR173 (TRBV4-2_CASSQDVRQGGYGYTF_TRBJ1-2/TRAV29/DV5_CAASDQAGTALIF_TRAJ15). Lastly, TCR174 (TRBV14_CASSLQGRRGDTQYF_BJ2-3/TRAV29/DV5_CAASAGAGSYQLTF_TRAJ7) was shown to react against RFX4-60 (Fig. 3). As outlined earlier, TRBV4-2 is among a number of specific TCR segments of interest in the context of narcolepsy, as genetic QTL locations modulating its usage are strongly associated with this condition^13^. Here we found two TCRs (TCR165 from one patient and TCR173 from one control) shared TRBV4-2, hence suggesting some relationship between CD4+ T cell autoreactivity in these two donors and TRBV4-2 via RFX4-86. Notably, no cross-reaction between RFX4 and HCRT/Pandemrix was detected, suggesting these TCRs are RFX4 specific (Fig. 3). TCR activation by RFX4 further suggests RFX4 is involved in T1N and controls.

**Table 3:** Enriched TCRαβ from RFX4 antigen specific CD4+ CD8− T cells.

D	P	Dx	BV	CDR3β	BJ	AV	CDR3α	AJ	C#	TCR	Note
900	95	P	28	CASSSSGQGGNSPLHF	1-6	19	CALSGDVYGGSQGNLIF	42	74	162	
21	86	P	12-3	CASSLRGLNYGYTF	1-2	41	CAVLNRDDKIIF	30	66	163	
948	86	P	4–2	CASSQEGGKETQYF	2-5	2	CAGPSGYSTLTF	11	52	164	NE
						6	CALDRGTDKLIF*	34	49	165	
21	95	P	2	CASSGQGSYEQYF	2-7	9-2	CALSSLWGEKLTF	48	32	166	
						12-1	CVVNMNFGGGADGLTF*	45	32	167	NE
901	86	C	5-6	CASSRGRVGESSYEQYF	2-7	12-1	CVVNIENAGNMLTF	39	28	168	NE
21	95	P	28	CASSFSGTRTGELFF	2-2	3	CAPGRGSSNTGKLIF	37	26	169	NE
						12-2	CAVMPNDYKLSF*	20	26	170	
5688	86	C	4-2	CASSNNYGYTF	1-2	38-2/DV8	CARPANSGNTPLVF	29	23	171	NE
21	86	P	5-6	CASSLGPRTPKGQYF	2-7	39	CAVENNNDMRF	43	20	172	
5688	86	C	4-2	CASSQDVRQGGYGYTF	1-2	29/DV5	CAASDQAGTALIF	15	9	173	
848	60	P	14	CASSLQGRRGDTQYF	2-3	29/DV5	CAASAGAGSYQLTF	28	7	174	

*D* donor, *P* peptide, *Dx* diagnosis, *C* control, *P* patient, *C#* cell count, *NE* not expressed.

*Alternative CDR3α.

## Discussion

The autoimmune hypothesis of narcolepsy arose more than 30 years ago based on the strong association of HLA-DQ0602 of the disorder, and although initially, evidence in support of this hypothesis was hard to demonstrate, recent data now suggest T cell involvement in the destruction of HCRT cells. In this work, we investigate whether RFX4, which is highly enriched in HCRT cells, might be a relevant autoantigen in T1N. This was done by testing for the presence of autoantibody and/or of DQ0602 restricted T cell reactivity against it.

Whether or not autoantibodies are involved in the pathophysiology of narcolepsy is controversial. Studies of autoantibodies directed against hypocretin and hypocretin receptors in narcolepsy patients have rendered mixed results^35–38^. Other groups have studied reactivity to other proteins expressed in HCRT cells, such as TRIB2, with similarly mixed results^31,41^. Furthermore, one recent study investigated A/H1N1 hemagglutinin antibody levels and affinity in vaccine-related T1N, concluding that these antibodies are unlikely to contribute to pathogenesis^86^. Our results show that autoantibodies against RFX4 are rare in T1N patients, with anti-RFX4_v3 autoantibody-like reactivity being significantly increased solely in two patients (1%), both vaccinated with Pandemrix. None of these patients were positive for anti-HCRTR2 autoantibodies^37^. For anti-RFX4_v1/v4 autoantibodies, ΔMFI^PE^ of subject(s) above cut-off could not be repeated and dot plots showed no obvious correlation between PE and GFP channels (Fig. 1), suggesting that anti-RFX4_v1/v4 autoantibody levels in these subjects were too low to be detected. Taken together, these findings support previous data from our lab and others^34^, supporting that autoantibodies are unlikely to be involved in the pathophysiology of narcolepsy. Further to this, our findings validate that high throughput flow cytometry-based autoantibody detection assay as a method for primary autoantibody screening is reliable, and not solely for surface antigens.

Next, we probed the presence of antigen specific CD4+ T cells by co-culturing PBMCs from early-onset and post-Pandemrix T1N patients with Pandemrix or a single RFX4 peptide. Although tetramer DQ0602 positive CD4+ T cells were not clearly clustered when stimulated with Pandemrix, they were observed sporadically in both patients and controls. For example, cells reactive against RFX4-36, -76, and -172 were identified in an early-onset patient (165), PP-N patient (900), and PP-C (023), respectively (Supplemental Fig. S3). The proportions of CD4+ T cells recognized by tetramer pDQ0602 in CD3+ T cell populations were very small (generally lower than 0.085%, most < 0.05%), except for one control (901), which also had high background (peptide NC, 0.12%) (Supplemental Fig. S3).

In probing for the presence of RFX4-antigen specific CD4+ T cells, we detected reactivity against RFX4-60, -86 and -95 in one EO-N, three PP-N patients and two controls. We therefore sought to determine the identity of these cells further and conducted single cell TCR sequencing of reactive cells. Analysis of frequently used TRAV/TRBV pairings revealed that although V gene segments are partially similar between patients and controls, they differ significantly between RFX4-86, -95 and -60. To probe the reactivity of specific TCRs to these three peptides, we expressed enriched TCRαβs in Jurkat 76 (J76)-NFATRE-luc cells co-cultured with RM3-DQ0602. One TCR (TCR165) from one patient but also another TCR (TCR173) from one healthy donor reactive against RFX4-86 used TRBV4-2, which has been strongly implicated in narcolepsy pathophysiology by our lab before through genetic analysis^13^.

Although the autoimmune etiology of T1N was recently solidified, the exact pathophysiology underlying this disorder still remains elusive. Our findings suggest that future work should be dedicated to investigating the exact role of RFX4 in T1N and how reactivity to this protein may impact HCRT neuronal death. Earlier studies found RFX4 restricted to testis^62^, however, later works also demonstrated a role for this transcription factor in brain development^65^. Thus, it is conceivable that not only HCRT neurons could be affected by autoantibodies or CD4+ T cells against RFX4, but also other cell types and tissues. Of note, however, the reactivity against this peptide was not sex-restricted and found in both males and females.

While it was feasible to conduct autoantibody screening in a large number of subjects, a limitation of our study is that not an equally high number was assessed for autoreactive CD4+ T cells due to the non-high-throughput and laborious protocol of competing binding assay and DQ0602 tetramer staining. Nevertheless, competing binding assay and DQ0602 tetramer staining assays were found to be feasible and reliable for screening autoantigen reactivity in T1N, so that these may now be extended to the study of more antigens and patients using new technologies that have higher throughput. Such additional experiments could for example use 10× compatible single cell analysis with DNA barcode labelling and pooling of tetramer reactivity for both strong and weak RFX4 to DQ0602. Single cell TCR sequencing and phenotyping of more RFX4 specific CD4+ and CD8+ T cells could elucidate the role of RFX4 in the pathophysiology of narcolepsy.

All in all, due to co-localization of RFX4 with hypocretin and highly enrichment specific to hypocretin neurons^34,45,54,55^, we hypothesized a role for RFX4 as an autoantigen in T1N, which is caused by loss of hypocretin (> 90%) and genetically associated with TRBV4-2 10. While the evidence we provide in this study suggests that autoantibodies against this peptide are rare and unlikely to be involved in the pathophysiology and onset of this condition, T cell reactivity against RFX4 peptides was sound in some narcolepsy patients, but also in healthy donors. Importantly, the enrichment of TRBV4-2 in one narcolepsy patient and one healthy donor suggests the involvement of RFX4 CD4+ T cell autoreactivity in these donors. Overall, some autoreactive CD4+ T cells directed against RFX4 have been detected in narcolepsy patients, but also in healthy donors.

## Supplementary Information

Supplementary Information 1.

Supplementary Information 2.

Supplementary Information 3.

Supplementary Information 4.

Supplementary Information 5.
