# Supplementary material for: T cell reactivity to regulatory factor X4 in type 1 narcolepsy
Source: Sci Rep. 2021 Apr 9;11:7841. doi: 10.1038/s41598-021-87481-8 (PMC8035403; doi:10.1038/s41598-021-87481-8)
Supplement: Supplementary file 5 — Supplementary Information 5. [file 41598_2021_87481_MOESM5_ESM.pdf]

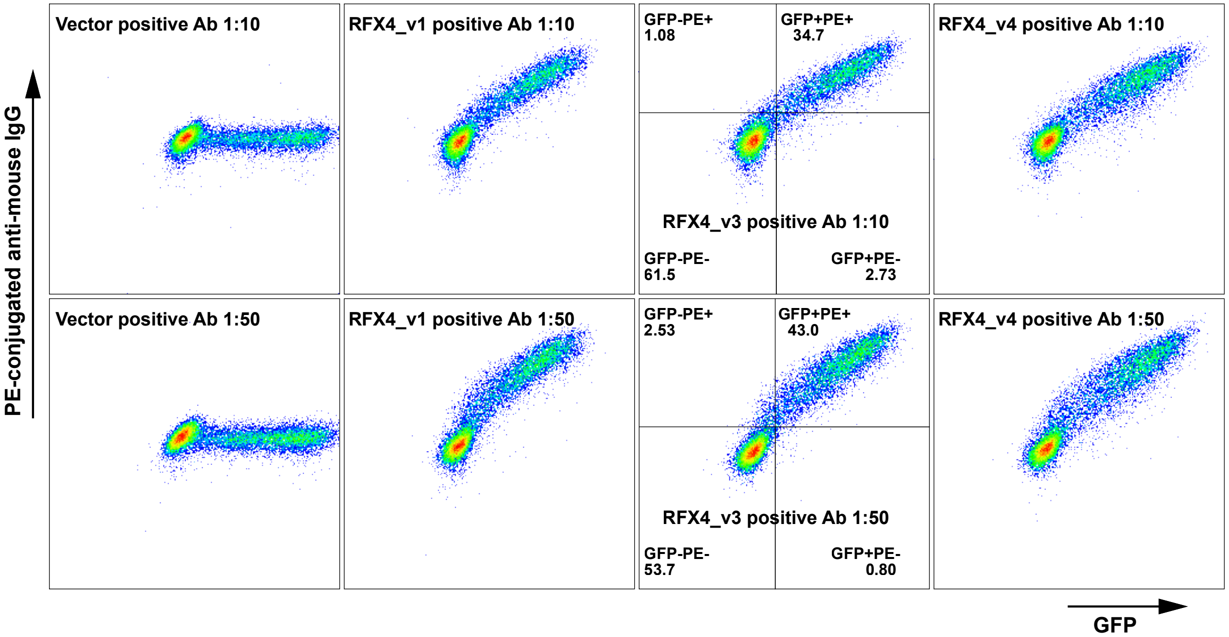


**Supplemental Fig. 1**. Staining control for flow cytometry-based autoantibody detection. HEK293T cells were transfected with RFX4_v1-GFP, RFX4_v3-GFP, RFX4_v4-GFP or pCMV6-AC-GFP and stained with positive mouse anti-RFX4 antibody at different dilution ratios and PE anti-mouse IgG (1:100) after culturing for 24 hours. Equal events were recorded and live single cells are shown for each construct and % of each quadrant population in live single cells for RFX4_v3 is shown. Ab, antibody. It was performed at least once. Plots were analyzed with FlowJo (version 10.0.8, Becton, Dickinson and Company).

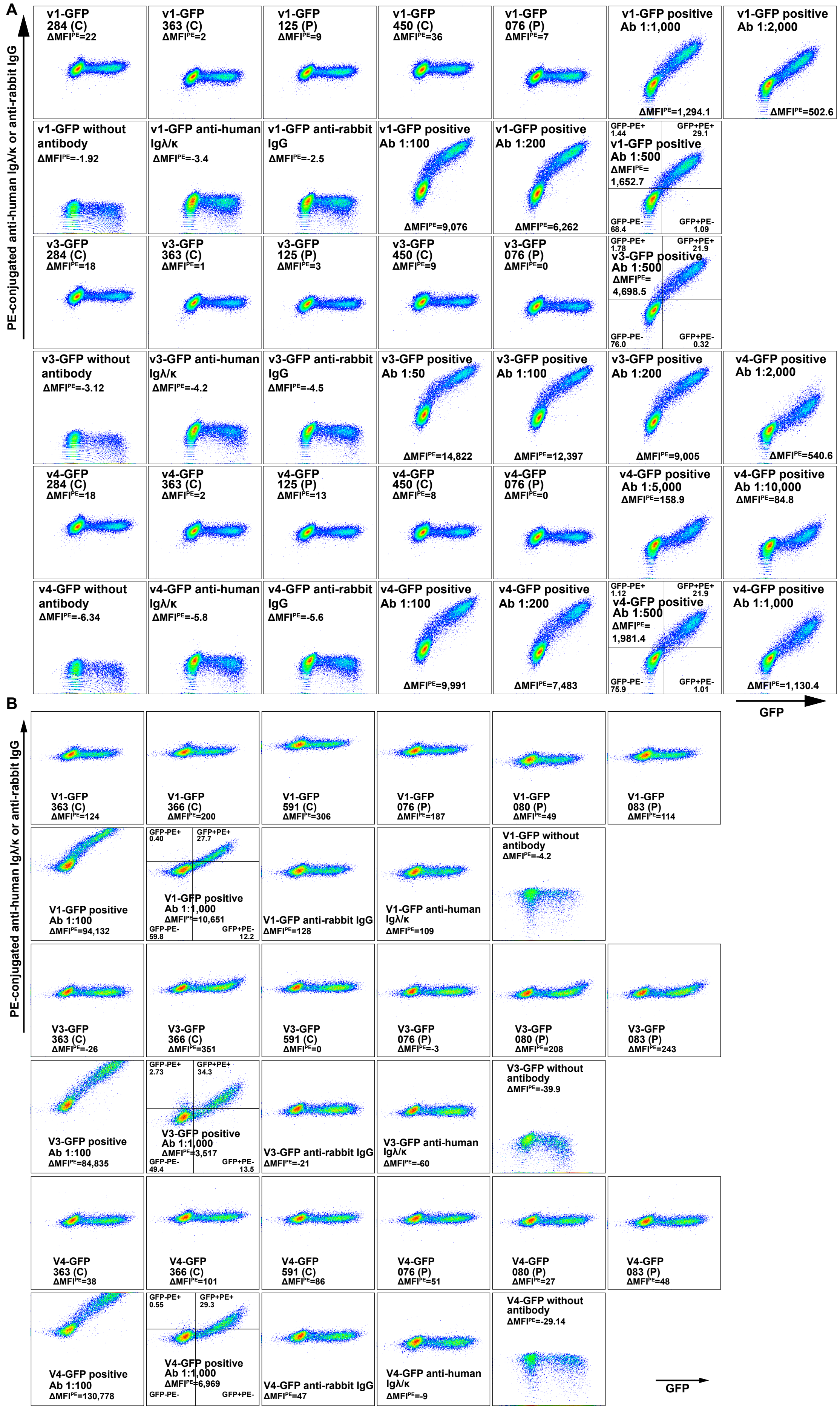
A
PE-conjugated anti-human Igλ/κ or anti-rabbit IgG
v1-GFP 284 (C) ΔMFI^PE=22
v1-GFP 363 (C) ΔMFI^PE=2
v1-GFP 125 (P) ΔMFI^PE=9
v1-GFP 450 (C) ΔMFI^PE=36
v1-GFP 076 (P) ΔMFI^PE=7
v1-GFP positive Ab 1:1,000 ΔMFI^PE=1,294.1
v1-GFP positive Ab 1:2,000 ΔMFI^PE=502.6
v1-GFP without antibody ΔMFI^PE=-1.92
v1-GFP anti-human Igλ/κ ΔMFI^PE=-3.4
v1-GFP anti-rabbit IgG ΔMFI^PE=-2.5
v1-GFP positive Ab 1:100 ΔMFI^PE=9,076
v1-GFP positive Ab 1:200 ΔMFI^PE=6,262
GFP-PE+ 1.44
GFP+PE+ 29.1
v1-GFP positive Ab 1:500 ΔMFI^PE= 1,652.7
GFP-PE- 68.4
GFP+PE- 1.09
v3-GFP 284 (C) ΔMFI^PE=18
v3-GFP 363 (C) ΔMFI^PE=1
v3-GFP 125 (P) ΔMFI^PE=3
v3-GFP 450 (C) ΔMFI^PE=9
v3-GFP 076 (P) ΔMFI^PE=0
GFP-PE+ 1.78
GFP+PE+ 21.9
v3-GFP positive Ab 1:500 ΔMFI^PE= 4,698.5
GFP-PE- 76.0
GFP+PE- 0.32
v3-GFP without antibody ΔMFI^PE=-3.12
v3-GFP anti-human Igλ/κ ΔMFI^PE=-4.2
v3-GFP anti-rabbit IgG ΔMFI^PE=-4.5
v3-GFP positive Ab 1:50 ΔMFI^PE=14,822
v3-GFP positive Ab 1:100 ΔMFI^PE=12,397
v3-GFP positive Ab 1:200 ΔMFI^PE=9,005
v4-GFP positive Ab 1:2,000 ΔMFI^PE=540.6
v4-GFP 284 (C) ΔMFI^PE=18
v4-GFP 363 (C) ΔMFI^PE=2
v4-GFP 125 (P) ΔMFI^PE=13
v4-GFP 450 (C) ΔMFI^PE=8
v4-GFP 076 (P) ΔMFI^PE=0
v4-GFP positive Ab 1:5,000 ΔMFI^PE=158.9
v4-GFP positive Ab 1:10,000 ΔMFI^PE=84.8
v4-GFP without antibody ΔMFI^PE=-6.34
v4-GFP anti-human Igλ/κ ΔMFI^PE=-5.8
v4-GFP anti-rabbit IgG ΔMFI^PE=-5.6
v4-GFP positive Ab 1:100 ΔMFI^PE=9,991
v4-GFP positive Ab 1:200 ΔMFI^PE=7,483
GFP-PE+ 1.12
GFP+PE+ 21.9
v4-GFP positive Ab 1:500 ΔMFI^PE= 1,981.4
GFP-PE- 75.9
GFP+PE- 1.01
v4-GFP positive Ab 1:1,000 ΔMFI^PE=1,130.4
GFP
B
PE-conjugated anti-human Igλ/κ or anti-rabbit IgG
V1-GFP 363 (C) ΔMFI^PE=124
V1-GFP 366 (C) ΔMFI^PE=200
V1-GFP 591 (C) ΔMFI^PE=306
V1-GFP 076 (P) ΔMFI^PE=187
V1-GFP 080 (P) ΔMFI^PE=49
V1-GFP 083 (P) ΔMFI^PE=114
V1-GFP positive Ab 1:100 ΔMFI^PE=94,132
GFP-PE+ 0.40
GFP+PE+ 27.7
V1-GFP positive Ab 1:1,000 ΔMFI^PE=10,651
GFP-PE- 59.8
GFP+PE- 12.2
V1-GFP anti-rabbit IgG ΔMFI^PE=128
V1-GFP anti-human Igλ/κ ΔMFI^PE=109
V1-GFP without antibody ΔMFI^PE=-4.2
V3-GFP 363 (C) ΔMFI^PE=-26
V3-GFP 366 (C) ΔMFI^PE=351
V3-GFP 591 (C) ΔMFI^PE=0
V3-GFP 076 (P) ΔMFI^PE=-3
V3-GFP 080 (P) ΔMFI^PE=208
V3-GFP 083 (P) ΔMFI^PE=243
V3-GFP positive Ab 1:100 ΔMFI^PE=84,835
GFP-PE+ 2.73
GFP+PE+ 34.3
V3-GFP positive Ab 1:1,000 ΔMFI^PE=3,517
GFP-PE- 49.4
GFP+PE- 13.5
V3-GFP anti-rabbit IgG ΔMFI^PE=-21
V3-GFP anti-human Igλ/κ ΔMFI^PE=-60
V3-GFP without antibody ΔMFI^PE=-39.9
V4-GFP 363 (C) ΔMFI^PE=38
V4-GFP 366 (C) ΔMFI^PE=101
V4-GFP 591 (C) ΔMFI^PE=86
V4-GFP 076 (P) ΔMFI^PE=51
V4-GFP 080 (P) ΔMFI^PE=27
V4-GFP 083 (P) ΔMFI^PE=48
V4-GFP positive Ab 1:100 ΔMFI^PE=130,778
GFP-PE+ 0.55
GFP+PE+ 29.3
V4-GFP positive Ab 1:1,000 ΔMFI^PE=6,969
GFP-PE-
GFP+PE-
V4-GFP anti-rabbit IgG ΔMFI^PE=47
V4-GFP anti-human Igλ/κ ΔMFI^PE=-9
V4-GFP without antibody ΔMFI^PE=-29.14
GFP

**Supplemental Fig. 2**. FACS dot plots of antibody detection (A) with repeat (B). HEK293T cells were transfected with RFX4_v1, v3 or v4-GFP and stained with human serum (1:20) or positive rabbit anti-RFX4 antibody, followed by PE anti-human Igλ/κ antibody (1:100) or PE anti-rabbit IgG antibody (1:100), respectively. Equal events were recorded and live single cells are shown for each variant. % of each quadrant population in live single cells for positive antibodies is shown. Sensitivity was tested using diluted positive antibody. HEK293T cells stained with only secondary antibody or without any antibody are negative controls. $\Delta MFI^{PE}$ = ($MFI^{PE}$ of $HEK293T^{GFP+}$) - ($MFI^{PE}$ of $HEK293T^{GFP-}$). Ab, antibody; EO-N, early onset patient; PP-N, post-Pandemrix patient; PP-C, post-Pandemrix control; O-C, other control. It was performed at least once. Plots were analyzed with FlowJo (version 10.0.8, Becton, Dickinson and Company).

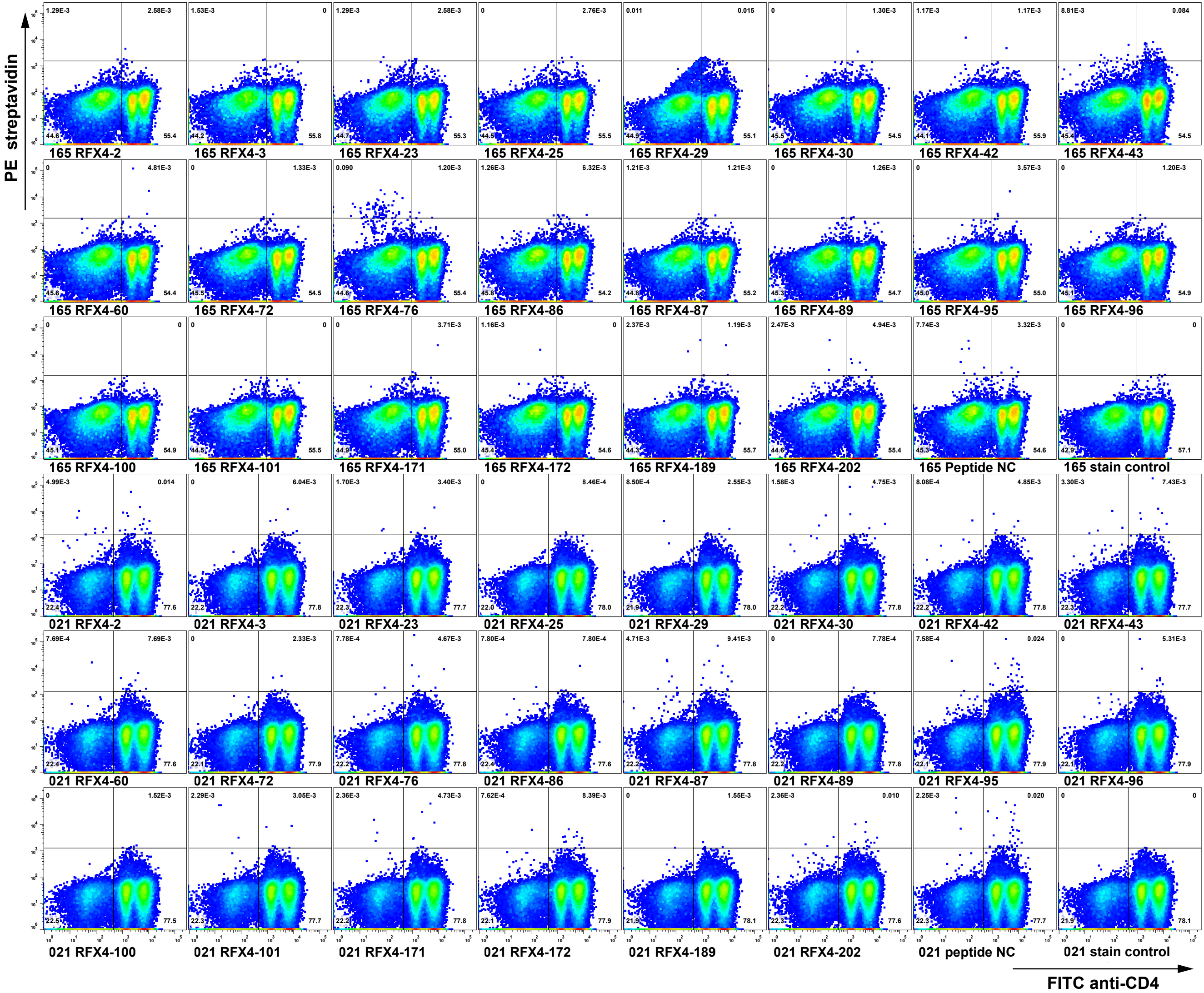

PE streptavidin
FITC anti-CD4
1.29E-3
2.58E-3
44.6
55.4
165 RFX4-2
1.53E-3
0
44.2
55.8
165 RFX4-3
1.29E-3
2.58E-3
44.7
55.3
165 RFX4-23
0
2.76E-3
44.5
55.5
165 RFX4-25
0.011
0.015
44.9
55.1
165 RFX4-29
0
1.30E-3
45.5
54.5
165 RFX4-30
1.17E-3
1.17E-3
44.1
55.9
165 RFX4-42
8.81E-3
0.084
45.4
54.5
165 RFX4-43
0
4.81E-3
45.6
54.4
165 RFX4-60
0
1.33E-3
45.5
54.5
165 RFX4-72
0.090
1.20E-3
44.6
55.4
165 RFX4-76
1.26E-3
6.32E-3
45.8
54.2
165 RFX4-86
1.21E-3
1.21E-3
44.8
55.2
165 RFX4-87
0
1.26E-3
45.3
54.7
165 RFX4-89
0
3.57E-3
45.0
55.0
165 RFX4-95
0
1.20E-3
45.1
54.9
165 RFX4-96
0
0
45.1
54.9
165 RFX4-100
0
0
44.5
55.5
165 RFX4-101
0
3.71E-3
44.9
55.0
165 RFX4-171
1.16E-3
0
45.4
54.6
165 RFX4-172
2.37E-3
1.19E-3
44.3
55.7
165 RFX4-189
2.47E-3
4.94E-3
44.6
55.4
165 RFX4-202
7.74E-3
3.32E-3
45.3
54.6
165 Peptide NC
0
0
42.9
57.1
165 stain control
4.99E-3
0.014
22.4
77.6
021 RFX4-2
0
6.04E-3
22.2
77.8
021 RFX4-3
1.70E-3
3.40E-3
22.3
77.7
021 RFX4-23
0
8.46E-4
22.0
78.0
021 RFX4-25
8.50E-4
2.55E-3
21.9
78.0
021 RFX4-29
1.58E-3
4.75E-3
22.2
77.8
021 RFX4-30
8.08E-4
4.85E-3
22.2
77.8
021 RFX4-42
3.30E-3
7.43E-3
22.3
77.7
021 RFX4-43
7.69E-4
7.69E-3
22.4
77.6
021 RFX4-60
0
2.33E-3
22.1
77.9
021 RFX4-72
7.78E-4
4.67E-3
22.2
77.8
021 RFX4-76
7.80E-4
7.80E-4
22.4
77.6
021 RFX4-86
4.71E-3
9.41E-3
22.2
77.8
021 RFX4-87
0
7.78E-4
22.2
77.8
021 RFX4-89
7.58E-4
0.024
22.1
77.9
021 RFX4-95
0
5.31E-3
22.1
77.9
021 RFX4-96
0
1.52E-3
22.5
77.5
021 RFX4-100
2.29E-3
3.05E-3
22.3
77.7
021 RFX4-101
2.36E-3
4.73E-3
22.2
77.8
021 RFX4-171
7.62E-4
8.39E-3
22.1
77.9
021 RFX4-172
0
1.55E-3
21.9
78.1
021 RFX4-189
2.36E-3
0.010
22.3
77.6
021 RFX4-202
2.25E-3
0.020
22.3
77.7
021 peptide NC
0
0
21.9
78.1
021 stain control

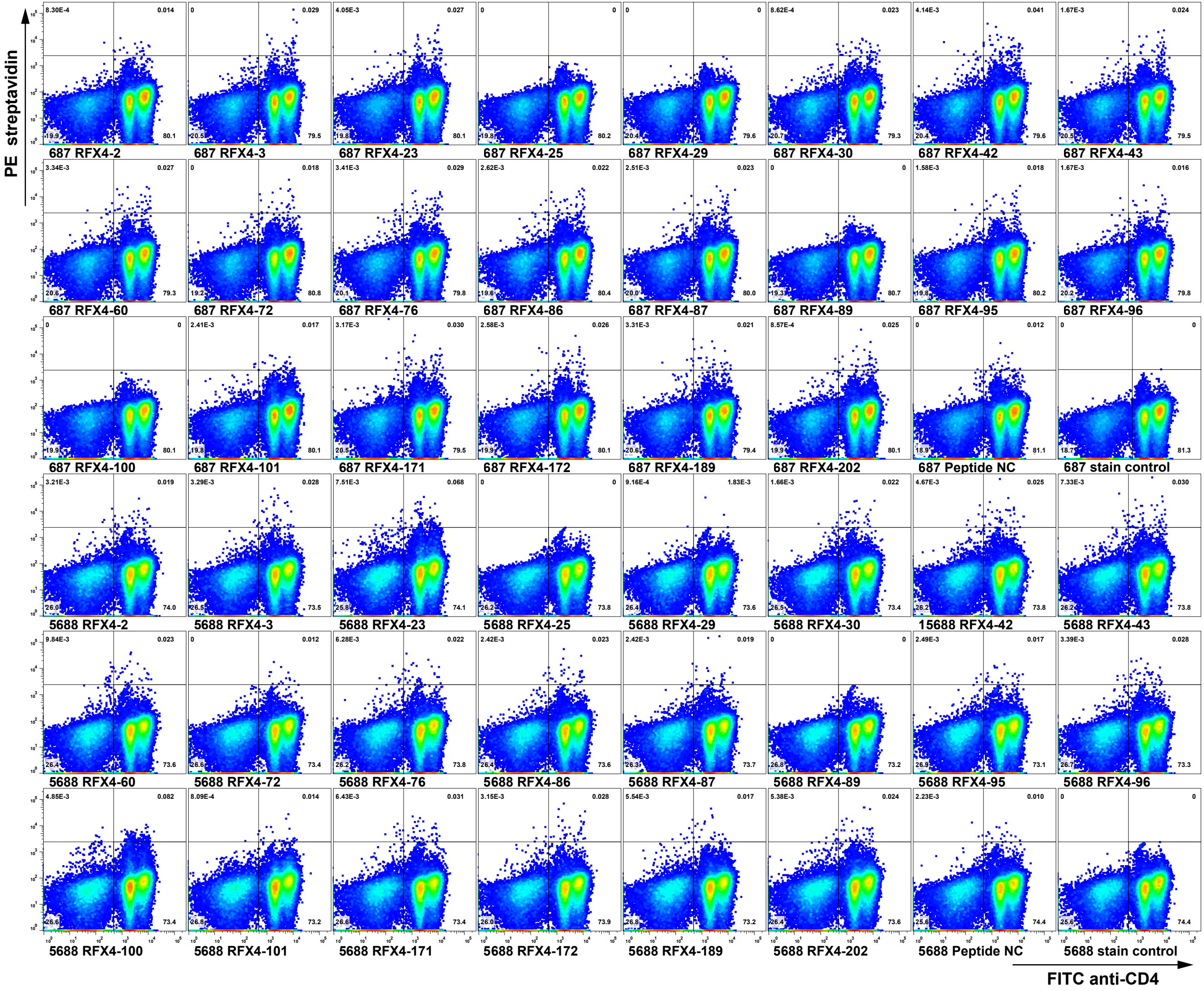
PE streptavidin
FITC anti-CD4
687 RFX4-2
687 RFX4-3
687 RFX4-23
687 RFX4-25
687 RFX4-29
687 RFX4-30
687 RFX4-42
687 RFX4-43
687 RFX4-60
687 RFX4-72
687 RFX4-76
687 RFX4-86
687 RFX4-87
687 RFX4-89
687 RFX4-95
687 RFX4-96
687 RFX4-100
687 RFX4-101
687 RFX4-171
687 RFX4-172
687 RFX4-189
687 RFX4-202
687 Peptide NC
687 stain control
5688 RFX4-2
5688 RFX4-3
5688 RFX4-23
5688 RFX4-25
5688 RFX4-29
5688 RFX4-30
15688 RFX4-42
5688 RFX4-43
5688 RFX4-60
5688 RFX4-72
5688 RFX4-76
5688 RFX4-86
5688 RFX4-87
5688 RFX4-89
5688 RFX4-95
5688 RFX4-96
5688 RFX4-100
5688 RFX4-101
5688 RFX4-171
5688 RFX4-172
5688 RFX4-189
5688 RFX4-202
5688 Peptide NC
5688 stain control

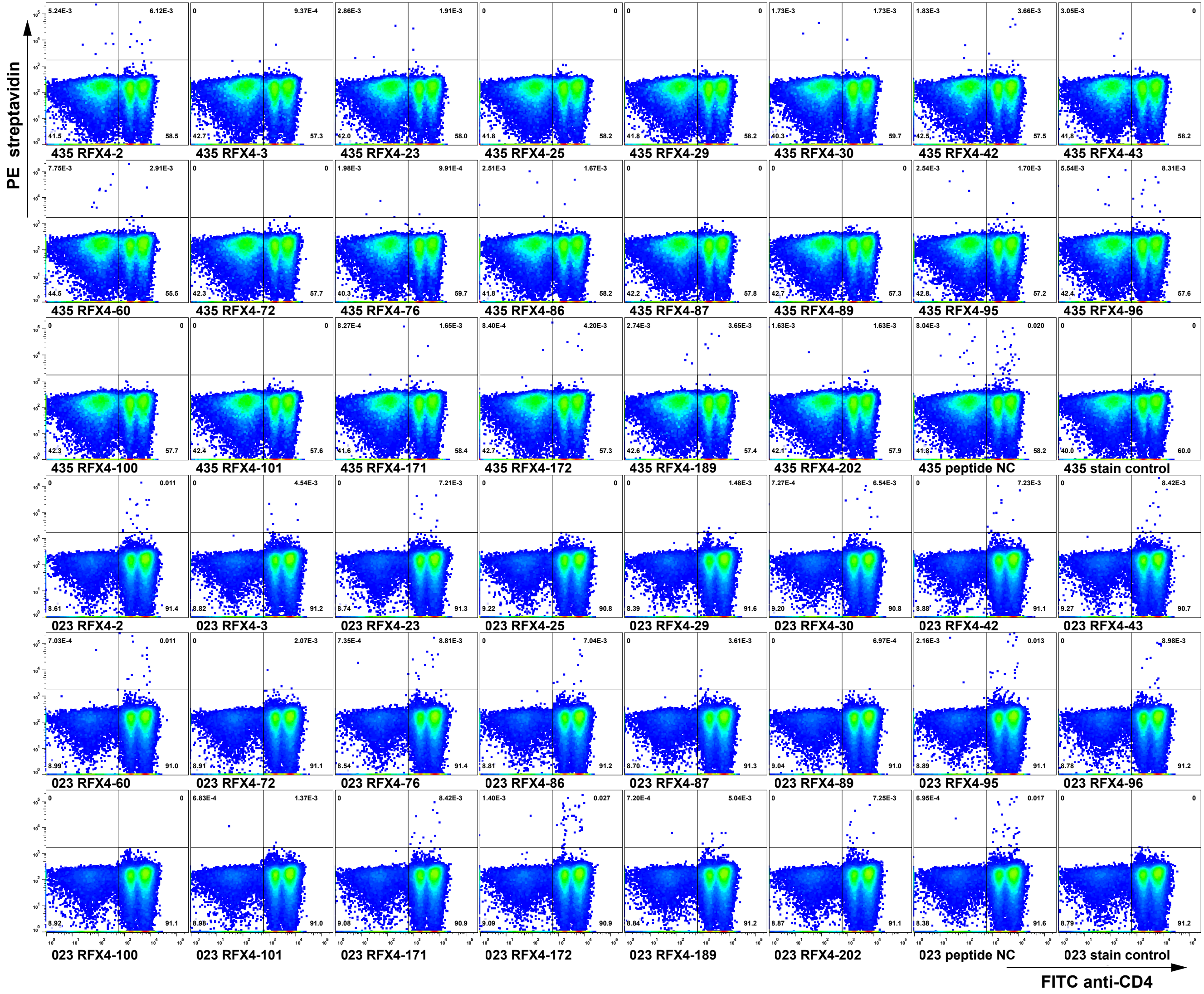
PE streptavidin
FITC anti-CD4
435 RFX4-2: 5.24E-3, 6.12E-3, 41.5, 58.5
435 RFX4-3: 0, 9.37E-4, 42.7, 57.3
435 RFX4-23: 2.86E-3, 1.91E-3, 42.0, 58.0
435 RFX4-25: 0, 0, 41.8, 58.2
435 RFX4-29: 0, 0, 41.8, 58.2
435 RFX4-30: 1.73E-3, 1.73E-3, 40.3, 59.7
435 RFX4-42: 1.83E-3, 3.66E-3, 42.5, 57.5
435 RFX4-43: 3.05E-3, 0, 41.8, 58.2
435 RFX4-60: 7.75E-3, 2.91E-3, 44.5, 55.5
435 RFX4-72: 0, 0, 42.3, 57.7
435 RFX4-76: 1.98E-3, 9.91E-4, 40.3, 59.7
435 RFX4-86: 2.51E-3, 1.67E-3, 41.8, 58.2
435 RFX4-87: 0, 0, 42.2, 57.8
435 RFX4-89: 0, 0, 42.7, 57.3
435 RFX4-95: 2.54E-3, 1.70E-3, 42.8, 57.2
435 RFX4-96: 5.54E-3, 8.31E-3, 42.4, 57.6
435 RFX4-100: 0, 0, 42.3, 57.7
435 RFX4-101: 0, 0, 42.4, 57.6
435 RFX4-171: 8.27E-4, 1.65E-3, 41.6, 58.4
435 RFX4-172: 8.40E-4, 4.20E-3, 42.7, 57.3
435 RFX4-189: 2.74E-3, 3.65E-3, 42.6, 57.4
435 RFX4-202: 1.63E-3, 1.63E-3, 42.1, 57.9
435 peptide NC: 8.04E-4, 0.020, 41.8, 58.2
435 stain control: 0, 0, 40.0, 60.0
023 RFX4-2: 0, 0.011, 8.61, 91.4
023 RFX4-3: 0, 4.54E-3, 8.82, 91.2
023 RFX4-23: 0, 7.21E-3, 8.74, 91.3
023 RFX4-25: 0, 0, 9.22, 90.8
023 RFX4-29: 0, 1.48E-3, 8.39, 91.6
023 RFX4-30: 7.27E-4, 6.54E-3, 9.20, 90.8
023 RFX4-42: 0, 7.23E-3, 8.88, 91.1
023 RFX4-43: 0, 8.42E-3, 9.27, 90.7
023 RFX4-60: 7.03E-4, 0.011, 8.99, 91.0
023 RFX4-72: 0, 2.07E-3, 8.91, 91.1
023 RFX4-76: 7.35E-4, 8.81E-3, 8.54, 91.4
023 RFX4-86: 0, 7.04E-3, 8.81, 91.2
023 RFX4-87: 0, 3.61E-3, 8.70, 91.3
023 RFX4-89: 0, 6.97E-4, 9.04, 91.0
023 RFX4-95: 2.16E-3, 0.013, 8.89, 91.1
023 RFX4-96: 0, 8.98E-3, 8.78, 91.2
023 RFX4-100: 0, 0, 8.92, 91.1
023 RFX4-101: 6.83E-4, 1.37E-3, 8.98, 91.0
023 RFX4-171: 0, 8.42E-3, 9.08, 90.9
023 RFX4-172: 1.40E-3, 0.027, 9.09, 90.9
023 RFX4-189: 7.20E-4, 5.04E-3, 8.84, 91.2
023 RFX4-202: 0, 7.25E-3, 8.87, 91.1
023 peptide NC: 6.95E-4, 0.017, 8.38, 91.6
023 stain control: 0, 0, 8.79, 91.2

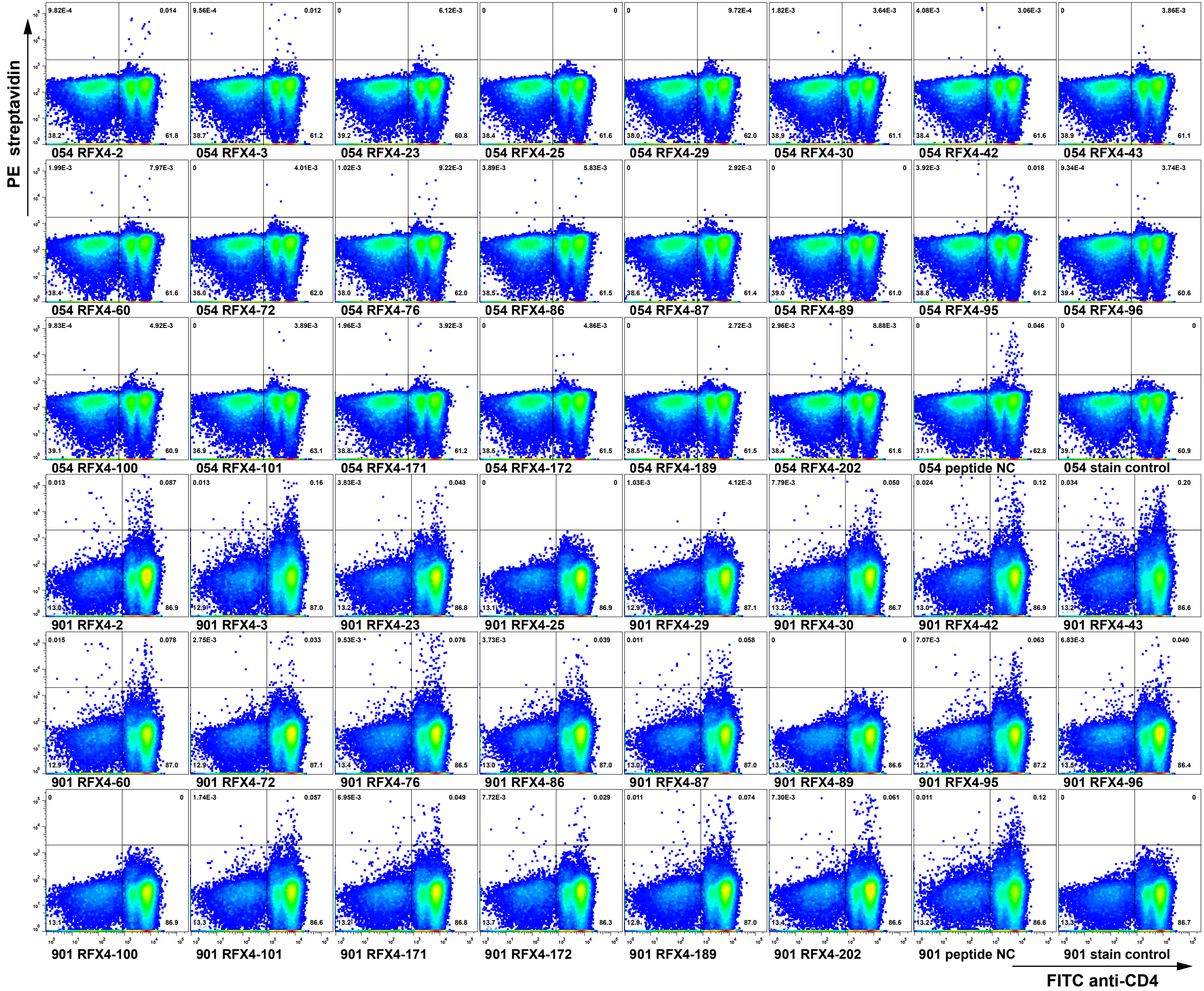
PE streptavidin
FITC anti-CD4
054 RFX4-2: 9.82E-4, 0.014, 38.2, 61.8
054 RFX4-3: 9.56E-4, 0.012, 38.7, 61.2
054 RFX4-23: 0, 6.12E-3, 39.2, 60.8
054 RFX4-25: 0, 0, 38.4, 61.6
054 RFX4-29: 0, 9.72E-4, 38.0, 62.0
054 RFX4-30: 1.82E-3, 3.64E-3, 38.9, 61.1
054 RFX4-42: 4.08E-3, 3.06E-3, 38.4, 61.6
054 RFX4-43: 0, 3.86E-3, 38.9, 61.1
054 RFX4-60: 1.99E-3, 7.97E-3, 38.4, 61.6
054 RFX4-72: 0, 4.01E-3, 38.0, 62.0
054 RFX4-76: 1.02E-3, 9.22E-3, 38.0, 62.0
054 RFX4-86: 3.89E-3, 5.83E-3, 38.5, 61.5
054 RFX4-87: 0, 2.92E-3, 38.6, 61.4
054 RFX4-89: 0, 0, 39.0, 61.0
054 RFX4-95: 3.92E-3, 0.018, 38.8, 61.2
054 RFX4-96: 9.34E-4, 3.74E-3, 39.4, 60.6
054 RFX4-100: 9.83E-4, 4.92E-3, 39.1, 60.9
054 RFX4-101: 0, 3.89E-3, 36.9, 63.1
054 RFX4-171: 1.96E-3, 3.92E-3, 38.8, 61.2
054 RFX4-172: 0, 4.86E-3, 38.5, 61.5
054 RFX4-189: 0, 2.72E-3, 38.5, 61.5
054 RFX4-202: 2.96E-3, 8.88E-3, 38.4, 61.6
054 peptide NC: 0, 0.046, 37.1, 62.8
054 stain control: 0, 0, 39.1, 60.9
901 RFX4-2: 0.013, 0.087, 13.0, 86.9
901 RFX4-3: 0.013, 0.16, 12.9, 87.0
901 RFX4-23: 3.83E-3, 0.043, 13.2, 86.8
901 RFX4-25: 0, 0, 13.1, 86.9
901 RFX4-29: 1.03E-3, 4.12E-3, 12.9, 87.1
901 RFX4-30: 7.79E-3, 0.050, 13.2, 86.7
901 RFX4-42: 0.024, 0.12, 13.0, 86.9
901 RFX4-43: 0.034, 0.20, 13.2, 86.6
901 RFX4-60: 0.015, 0.078, 12.9, 87.0
901 RFX4-72: 2.75E-3, 0.033, 12.9, 87.1
901 RFX4-76: 9.53E-3, 0.076, 13.4, 86.5
901 RFX4-86: 3.73E-3, 0.039, 13.0, 87.0
901 RFX4-87: 0.011, 0.058, 13.0, 87.0
901 RFX4-89: 0, 0, 13.4, 86.6
901 RFX4-95: 7.07E-3, 0.063, 12.7, 87.2
901 RFX4-96: 6.83E-3, 0.040, 13.5, 86.4
901 RFX4-100: 0, 0, 13.1, 86.9
901 RFX4-101: 1.74E-3, 0.057, 13.3, 86.6
901 RFX4-171: 6.95E-3, 0.049, 13.2, 86.8
901 RFX4-172: 7.72E-3, 0.029, 13.7, 86.3
901 RFX4-189: 0.011, 0.074, 12.9, 87.0
901 RFX4-202: 7.30E-3, 0.061, 13.4, 86.6
901 peptide NC: 0.011, 0.12, 13.2, 86.6
901 stain control: 0, 0, 13.3, 86.7

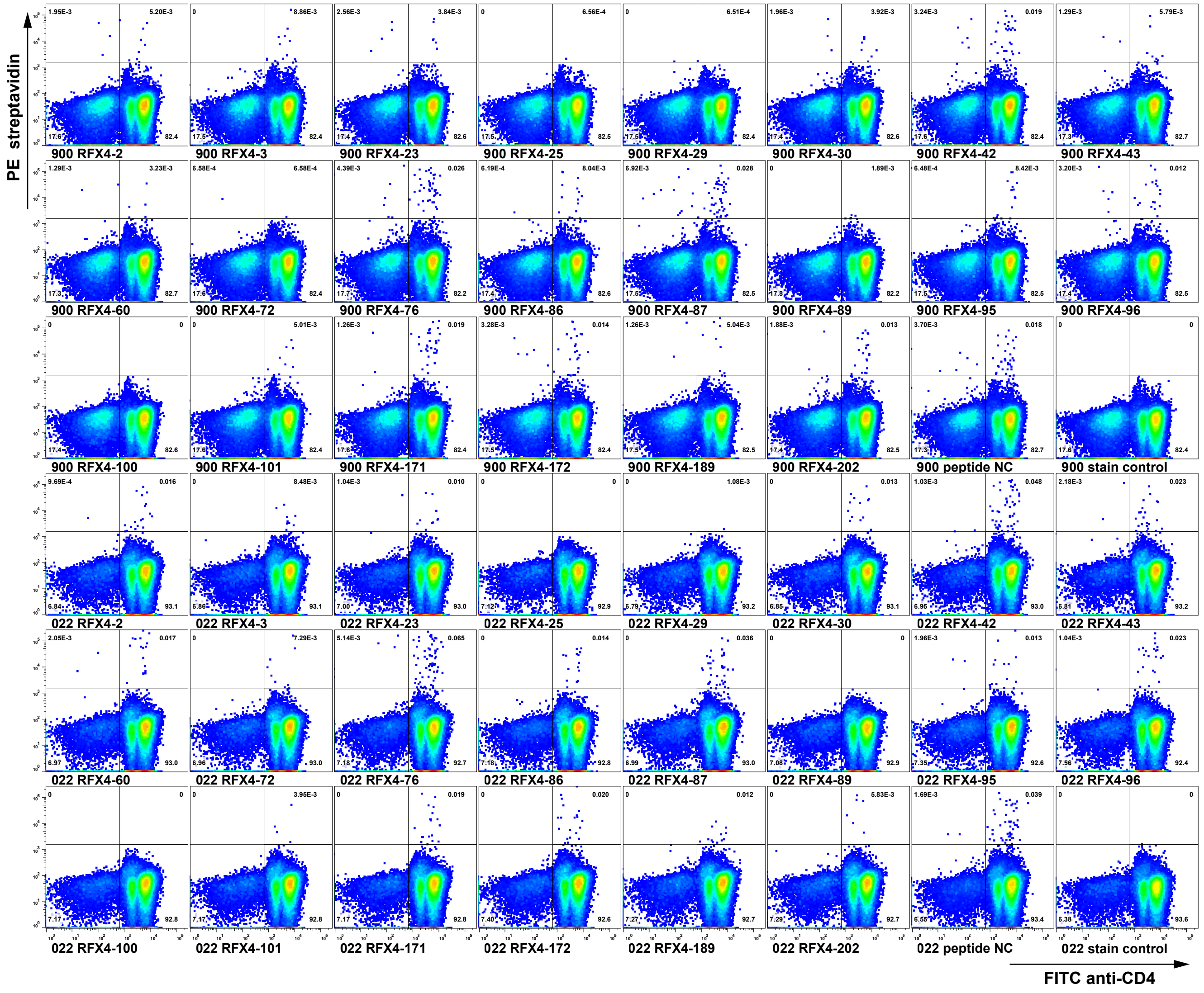

PE streptavidin
FITC anti-CD4
900 RFX4-2
900 RFX4-3
900 RFX4-23
900 RFX4-25
900 RFX4-29
900 RFX4-30
900 RFX4-42
900 RFX4-43
900 RFX4-60
900 RFX4-72
900 RFX4-76
900 RFX4-86
900 RFX4-87
900 RFX4-89
900 RFX4-95
900 RFX4-96
900 RFX4-100
900 RFX4-101
900 RFX4-171
900 RFX4-172
900 RFX4-189
900 RFX4-202
900 peptide NC
900 stain control
022 RFX4-2
022 RFX4-3
022 RFX4-23
022 RFX4-25
022 RFX4-29
022 RFX4-30
022 RFX4-42
022 RFX4-43
022 RFX4-60
022 RFX4-72
022 RFX4-76
022 RFX4-86
022 RFX4-87
022 RFX4-89
022 RFX4-95
022 RFX4-96
022 RFX4-100
022 RFX4-101
022 RFX4-171
022 RFX4-172
022 RFX4-189
022 RFX4-202
022 peptide NC
022 stain control

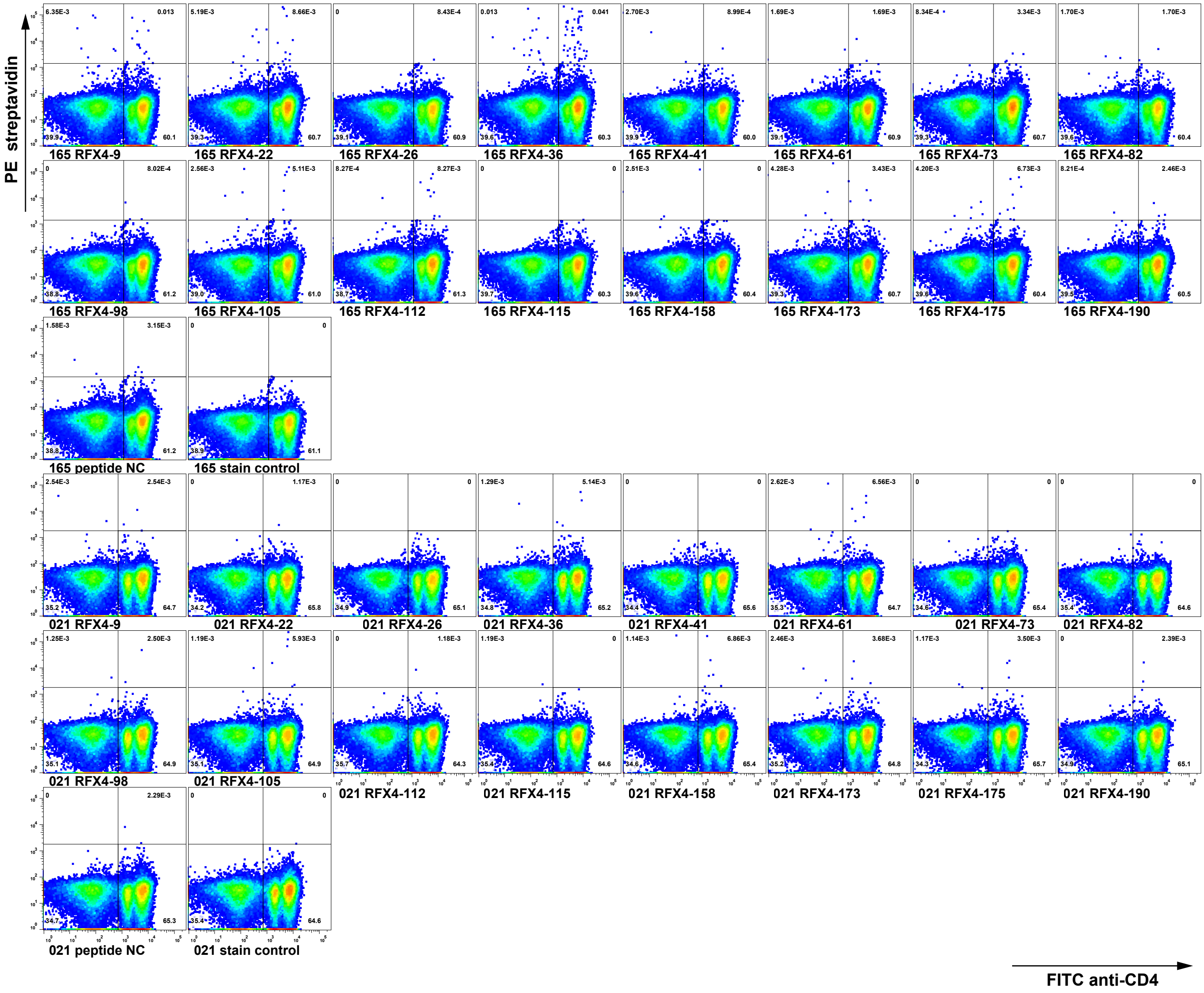

PE streptavidin
FITC anti-CD4
165 RFX4-9
6.35E-3 0.013 39.9 60.1
165 RFX4-22
5.19E-3 8.66E-3 39.3 60.7
165 RFX4-26
0 8.43E-4 39.1 60.9
165 RFX4-36
0.013 0.041 39.6 60.3
165 RFX4-41
2.70E-3 8.99E-4 39.9 60.0
165 RFX4-61
1.69E-3 1.69E-3 39.1 60.9
165 RFX4-73
8.34E-4 3.34E-3 39.3 60.7
165 RFX4-82
1.70E-3 1.70E-3 39.6 60.4
165 RFX4-98
0 8.02E-4 38.8 61.2
165 RFX4-105
2.56E-3 5.11E-3 39.0 61.0
165 RFX4-112
8.27E-4 8.27E-3 38.7 61.3
165 RFX4-115
0 0 39.7 60.3
165 RFX4-158
2.51E-3 0 39.6 60.4
165 RFX4-173
4.28E-3 3.43E-3 39.3 60.7
165 RFX4-175
4.20E-3 6.73E-3 39.6 60.4
165 RFX4-190
8.21E-4 2.46E-3 39.5 60.5
165 peptide NC
1.58E-3 3.15E-3 38.8 61.2
165 stain control
0 0 38.9 61.1
021 RFX4-9
2.54E-3 2.54E-3 35.2 64.7
021 RFX4-22
0 1.17E-3 34.2 65.8
021 RFX4-26
0 0 34.9 65.1
021 RFX4-36
1.29E-3 5.14E-3 34.8 65.2
021 RFX4-41
0 0 34.4 65.6
021 RFX4-61
2.62E-3 6.56E-3 35.3 64.7
021 RFX4-73
0 0 34.6 65.4
021 RFX4-82
0 0 35.4 64.6
021 RFX4-98
1.25E-3 2.50E-3 35.1 64.9
021 RFX4-105
1.19E-3 5.93E-3 35.1 64.9
021 RFX4-112
0 1.18E-3 35.7 64.3
021 RFX4-115
1.19E-3 0 35.4 64.6
021 RFX4-158
1.14E-3 6.86E-3 34.6 65.4
021 RFX4-173
2.46E-3 3.68E-3 35.2 64.8
021 RFX4-175
1.17E-3 3.50E-3 34.3 65.7
021 RFX4-190
0 2.39E-3 34.9 65.1
021 peptide NC
0 2.29E-3 34.7 65.3
021 stain control
0 0 35.4 64.6

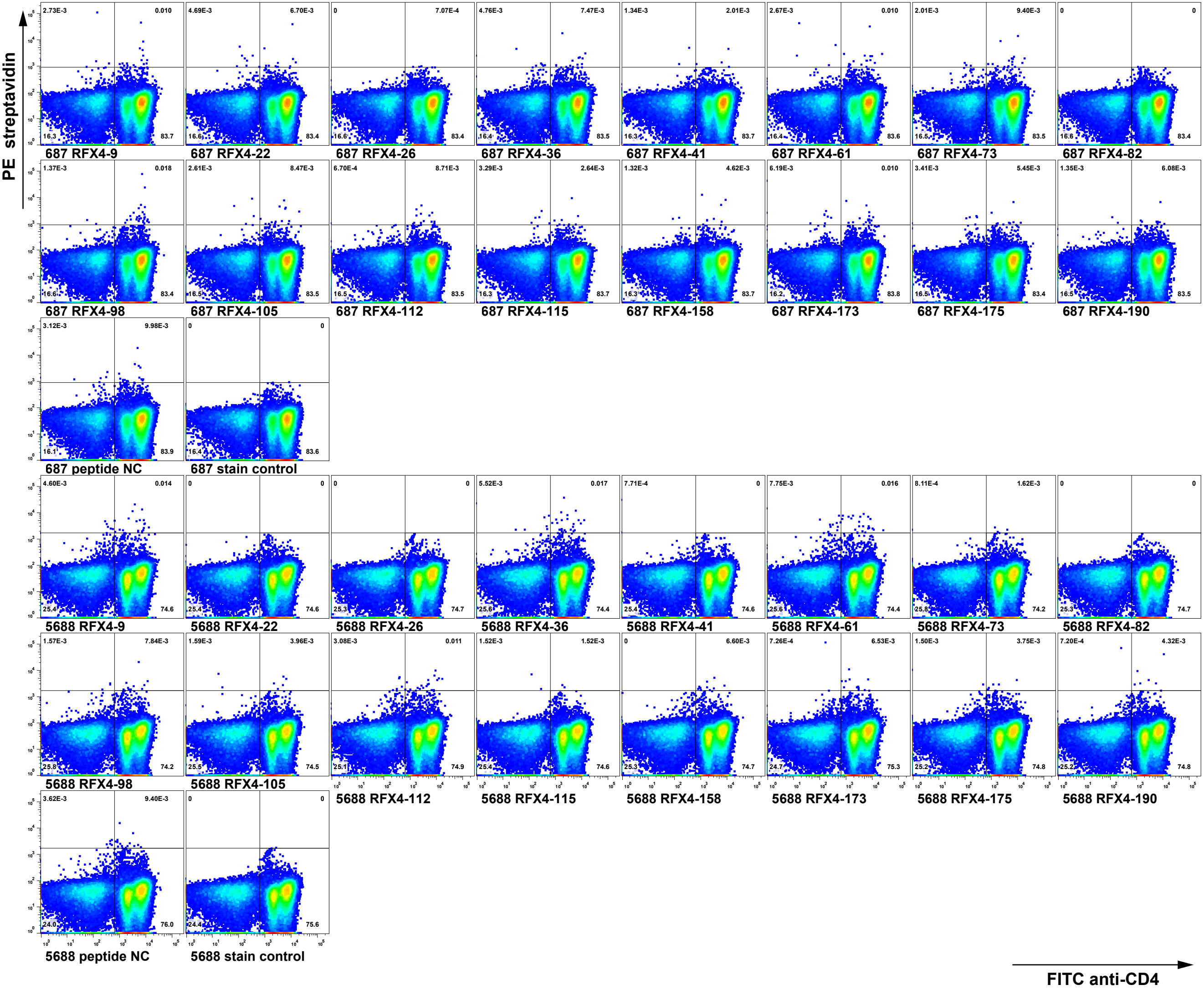
PE streptavidin
687 RFX4-9
687 RFX4-22
687 RFX4-26
687 RFX4-36
687 RFX4-41
687 RFX4-61
687 RFX4-73
687 RFX4-82
687 RFX4-98
687 RFX4-105
687 RFX4-112
687 RFX4-115
687 RFX4-158
687 RFX4-173
687 RFX4-175
687 RFX4-190
687 peptide NC
687 stain control
5688 RFX4-9
5688 RFX4-22
5688 RFX4-26
5688 RFX4-36
5688 RFX4-41
5688 RFX4-61
5688 RFX4-73
5688 RFX4-82
5688 RFX4-98
5688 RFX4-105
5688 RFX4-112
5688 RFX4-115
5688 RFX4-158
5688 RFX4-173
5688 RFX4-175
5688 RFX4-190
5688 peptide NC
5688 stain control
FITC anti-CD4

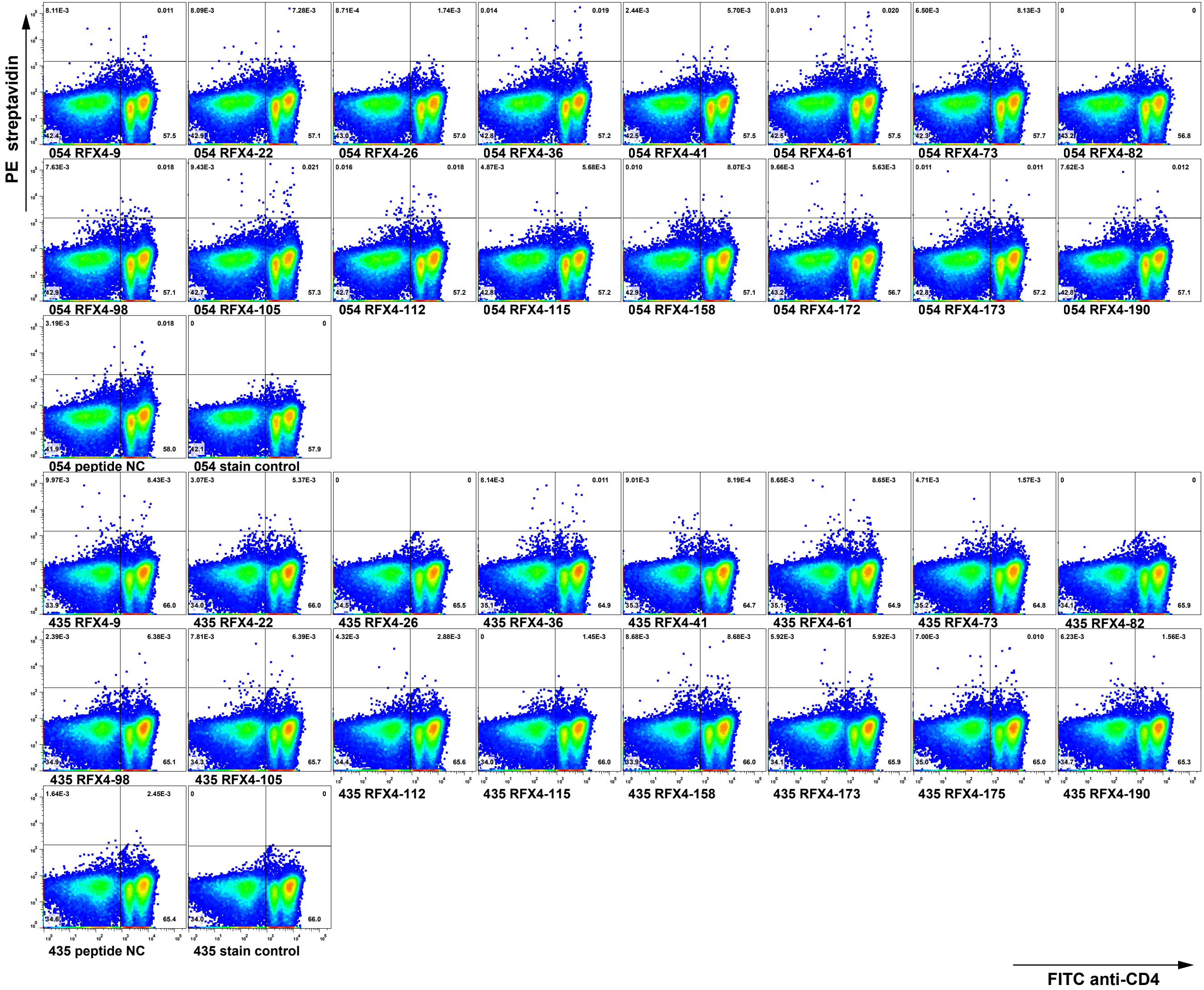
PE streptavidin
FITC anti-CD4
054 RFX4-9
054 RFX4-22
054 RFX4-26
054 RFX4-36
054 RFX4-41
054 RFX4-61
054 RFX4-73
054 RFX4-82
054 RFX4-98
054 RFX4-105
054 RFX4-112
054 RFX4-115
054 RFX4-158
054 RFX4-172
054 RFX4-173
054 RFX4-190
054 peptide NC
054 stain control
435 RFX4-9
435 RFX4-22
435 RFX4-26
435 RFX4-36
435 RFX4-41
435 RFX4-61
435 RFX4-73
435 RFX4-82
435 RFX4-98
435 RFX4-105
435 RFX4-112
435 RFX4-115
435 RFX4-158
435 RFX4-173
435 RFX4-175
435 RFX4-190
435 peptide NC
435 stain control

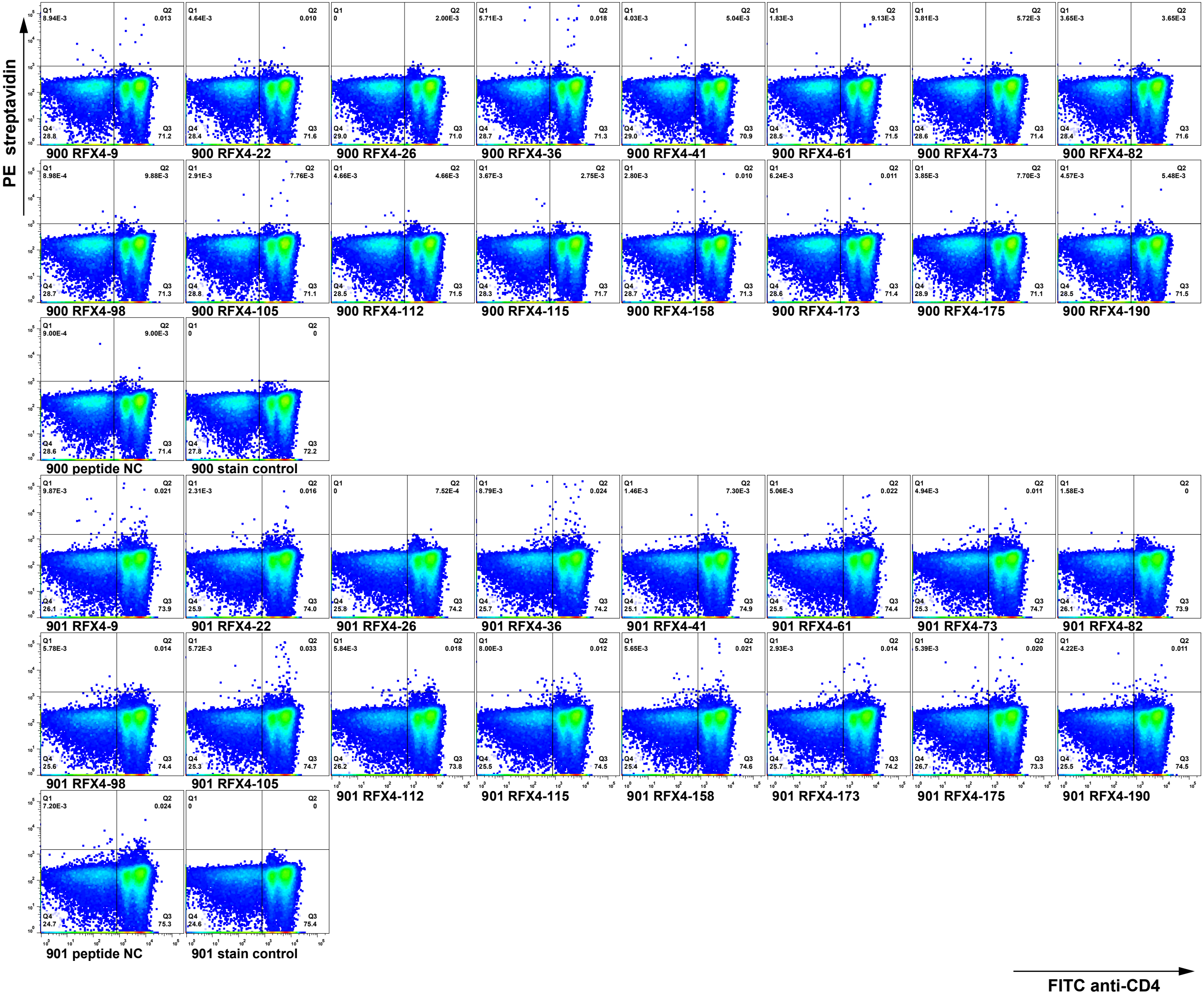
PE streptavidin
FITC anti-CD4
900 RFX4-9
900 RFX4-22
900 RFX4-26
900 RFX4-36
900 RFX4-41
900 RFX4-61
900 RFX4-73
900 RFX4-82
900 RFX4-98
900 RFX4-105
900 RFX4-112
900 RFX4-115
900 RFX4-158
900 RFX4-173
900 RFX4-175
900 RFX4-190
900 peptide NC
900 stain control
901 RFX4-9
901 RFX4-22
901 RFX4-26
901 RFX4-36
901 RFX4-41
901 RFX4-61
901 RFX4-73
901 RFX4-82
901 RFX4-98
901 RFX4-105
901 RFX4-112
901 RFX4-115
901 RFX4-158
901 RFX4-173
901 RFX4-175
901 RFX4-190
901 peptide NC
901 stain control

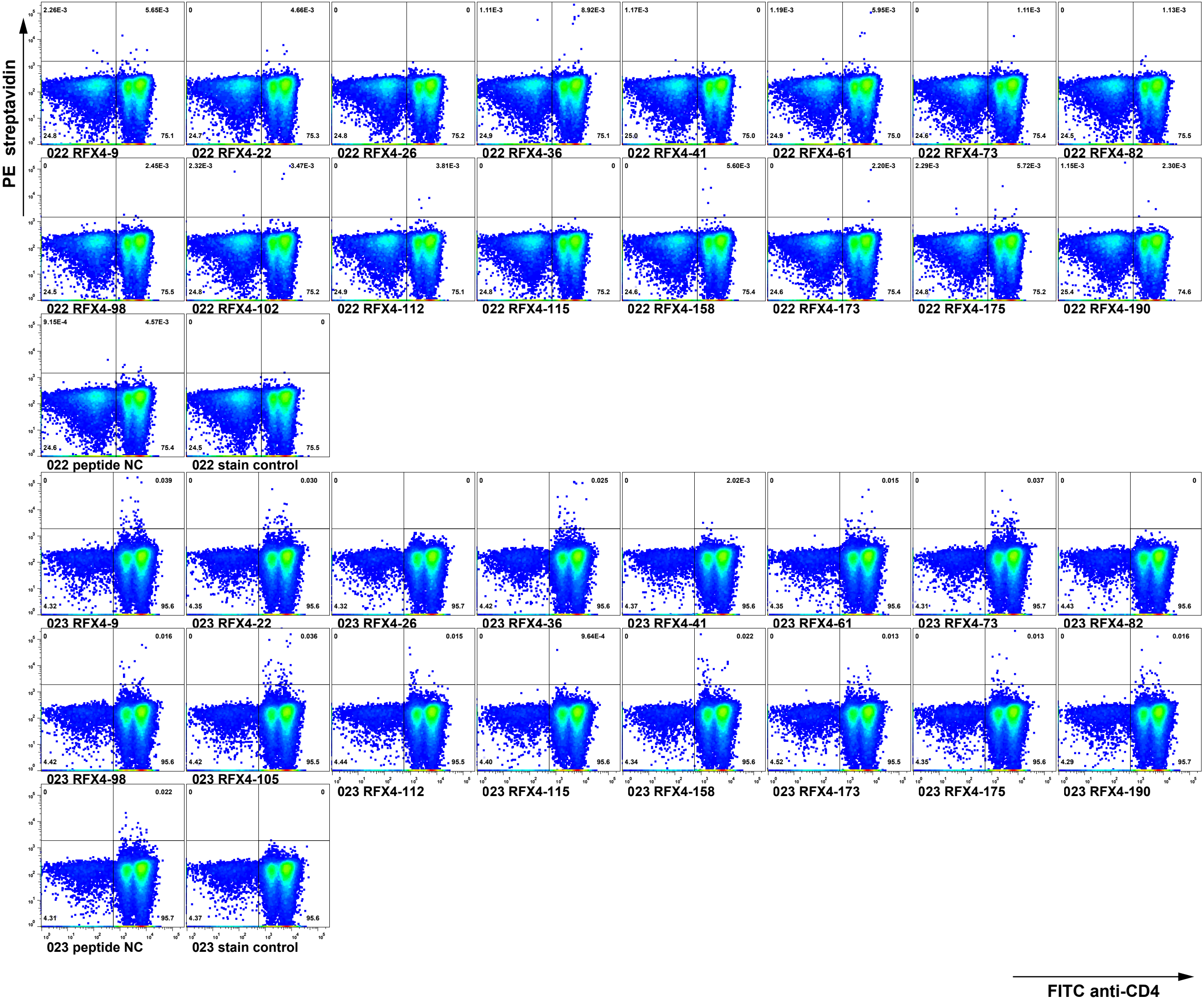

PE streptavidin
FITC anti-CD4
022 RFX4-9
2.26E-3 5.65E-3 24.8 75.1
022 RFX4-22
0 4.66E-3 24.7 75.3
022 RFX4-26
0 0 24.8 75.2
022 RFX4-36
1.11E-3 8.92E-3 24.9 75.1
022 RFX4-41
1.17E-3 0 25.0 75.0
022 RFX4-61
1.19E-3 5.95E-3 24.9 75.0
022 RFX4-73
0 1.11E-3 24.6 75.4
022 RFX4-82
0 1.13E-3 24.5 75.5
022 RFX4-98
0 2.45E-3 24.5 75.5
022 RFX4-102
2.32E-3 3.47E-3 24.8 75.2
022 RFX4-112
0 3.81E-3 24.9 75.1
022 RFX4-115
0 0 24.8 75.2
022 RFX4-158
0 5.60E-3 24.6 75.4
022 RFX4-173
0 2.20E-3 24.6 75.4
022 RFX4-175
2.29E-3 5.72E-3 24.8 75.2
022 RFX4-190
1.15E-3 2.30E-3 25.4 74.6
022 peptide NC
9.15E-4 4.57E-3 24.6 75.4
022 stain control
0 0 24.5 75.5
023 RFX4-9
0 0.039 4.32 95.6
023 RFX4-22
0 0.030 4.35 95.6
023 RFX4-26
0 0 4.32 95.7
023 RFX4-36
0 0.025 4.42 95.6
023 RFX4-41
0 2.02E-3 4.37 95.6
023 RFX4-61
0 0.015 4.35 95.6
023 RFX4-73
0 0.037 4.31 95.7
023 RFX4-82
0 0 4.43 95.6
023 RFX4-98
0 0.016 4.42 95.6
023 RFX4-105
0 0.036 4.42 95.5
023 RFX4-112
0 0.015 4.44 95.5
023 RFX4-115
0 9.64E-4 4.40 95.6
023 RFX4-158
0 0.022 4.34 95.6
023 RFX4-173
0 0.013 4.52 95.5
023 RFX4-175
0 0.013 4.35 95.6
023 RFX4-190
0 0.016 4.29 95.7
023 peptide NC
0 0.022 4.31 95.7
023 stain control
0 0 4.37 95.6

**Supplemental Fig. 3**. FACS dot plots of tetramer DQ0602-RFX4 staining with Pandemrix stimulation. PBMCs were stimulated with 100 ng/ml (final concentration of HA) of Pandemrix at $2.5\times10^6$ cells/ml for 10 days at 37°C, 5% $CO_2$. 20 IU/ml IL2 was supplemented from the $8^{th}$ to $10^{th}$ day. Cultured cells were incubated with PE-labelled tetramer DQ0602-RFX4 peptide for 90 minutes at 37°C, 5% $CO_2$, followed by staining with fluorescence labeled anti-CD3/CD4/CD8 antibodies. PI was added before FACS running. A quadrant gate was set according to staining with only anti-CD3/CD4/CD8 antibodies without PE-streptavidin (stain control) for each subject. Live single CD3+ T cells with percentage of each quadrant population are shown. Peptide NC indicated that cells were incubated with PE-tetramer DQ0602 without peptide loading. It was performed once. Plots were analyzed with FlowJo (version 10.0.8, Becton, Dickinson and Company).

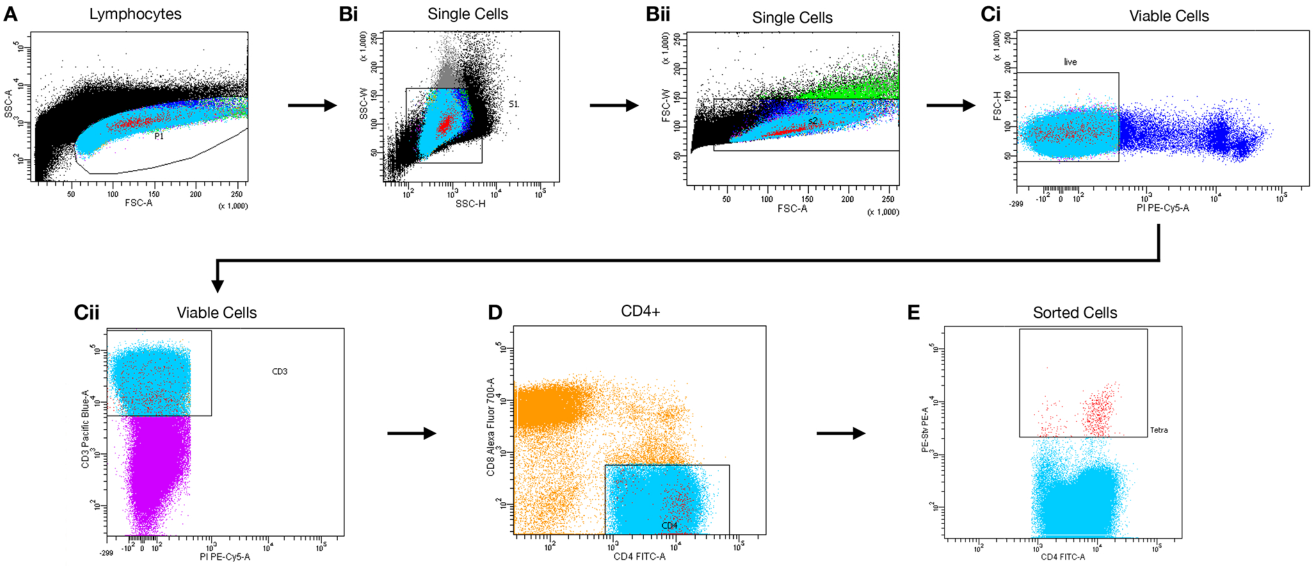


**Supplemental Fig. 4**. Sorting gate strategy of RFX4 antigen specific T cells. A. Lymphocytes were selected using forward (FSC-A) and side scatter (SSC-A) detectors. Bi. Single cells gating was conducted by parameters SSC-W and SSC-H and Bii. FSC-W and FSC-A. Ci. Viable cells were gated by the parameters FSC-H and Propidium iodide (PI) and Cii. CD3+ and PI. D. CD4+ cells were selected by the gating parameters CD4+ and CD8+. E. Final sorted cells were gated by PE Streptavidin and CD4+. Plots were analyzed with FlowJo (version 10.0.8, Becton, Dickinson and Company).

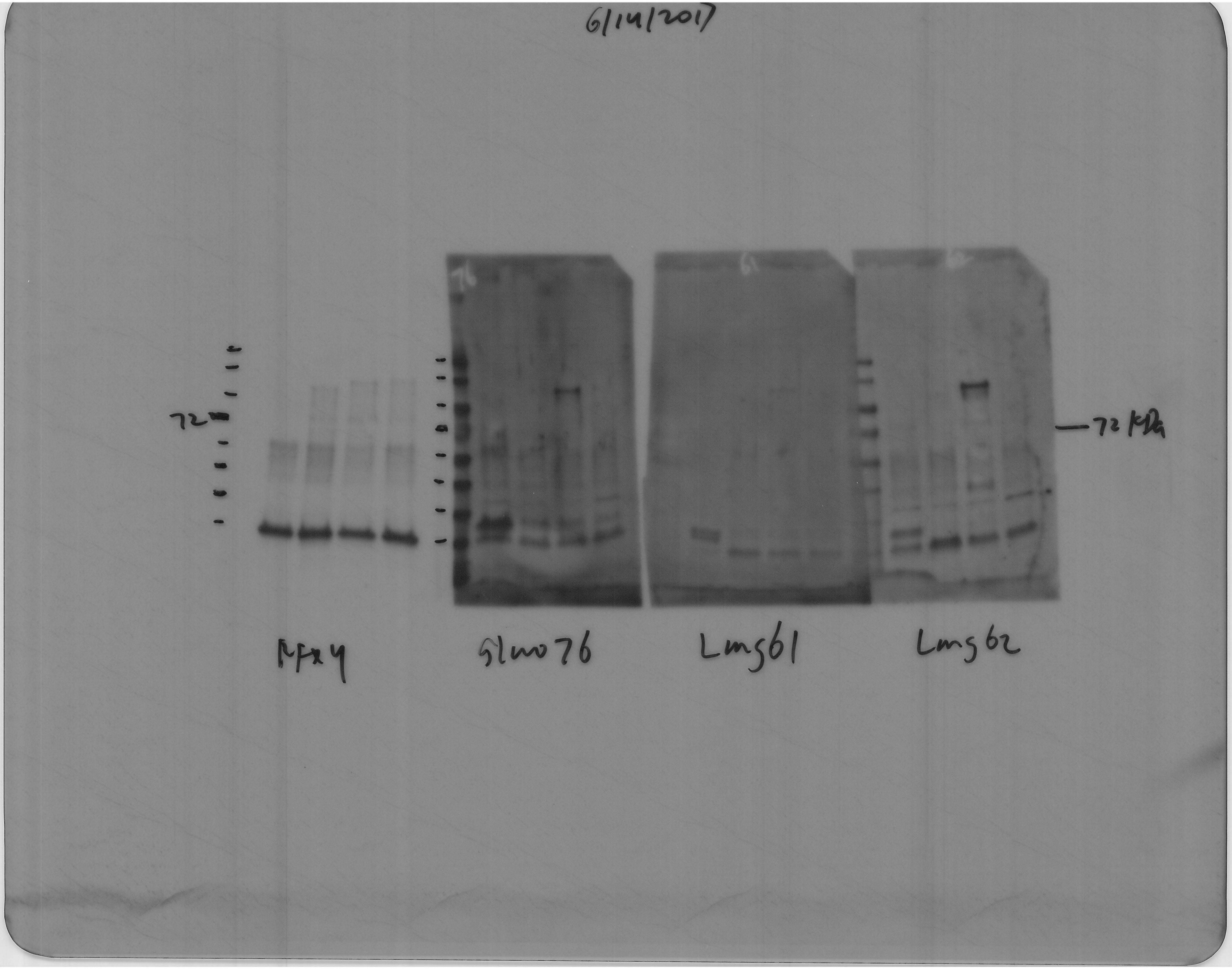
6/14/2017
72
72 kDa
Lmg61
Lmg62

**Supplemental Fig. 5**. The full-length gel corresponding to Fig. 1 (D). HEK293T cells expressing GFP-tagged RFX4_v1, v3, v4 and vector were lysed and anti-tGFP-conjugated agarose beads were incubated with supernatant for Co-IP. Cell lysates were loaded into two 10-lane gels and proteins were transferred to two polyvinylidene difluoride (PVDF) membranes. Each membrane was cut into two pieces for incubation with different sera and secondary antibodies. All four pieces of membranes were developed in one cassette for the same exposure. It was performed once. Image was developed with a tablet processor (Item# SRX-101A, Konica).

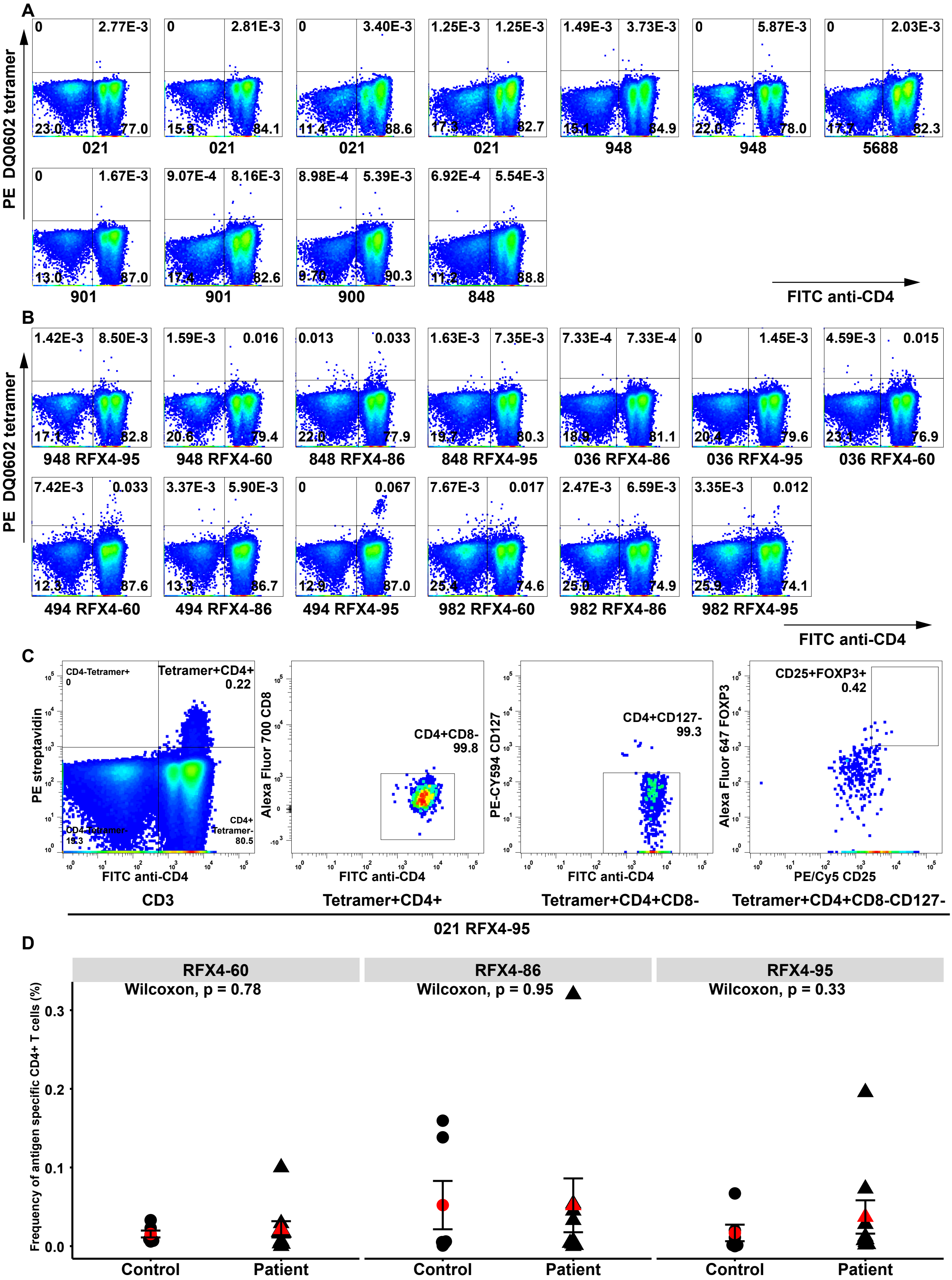

A
PE DQ0602 tetramer
FITC anti-CD4
021
948
5688
901
900
848
B
948 RFX4-95
948 RFX4-60
848 RFX4-86
848 RFX4-95
036 RFX4-86
036 RFX4-95
036 RFX4-60
494 RFX4-60
494 RFX4-86
494 RFX4-95
982 RFX4-60
982 RFX4-86
982 RFX4-95
C
CD4-Tetramer+ 0
Tetramer+CD4+ 0.22
CD4-Tetramer- 15.3
CD4+ Tetramer- 80.5
PE streptavidin
CD4+CD8- 99.8
Alexa Fluor 700 CD8
CD4+CD127- 99.3
PE-CY594 CD127
CD25+FOXP3+ 0.42
Alexa Fluor 647 FOXP3
PE/Cy5 CD25
CD3
Tetramer+CD4+
Tetramer+CD4+CD8-
Tetramer+CD4+CD8-CD127-
021 RFX4-95
D
RFX4-60
Wilcoxon, p = 0.78
RFX4-86
Wilcoxon, p = 0.95
RFX4-95
Wilcoxon, p = 0.33
Frequency of antigen specific CD4+ T cells (%)
Control
Patient

**Supplemental Fig. 6**. Dot plots of tetramer staining and frequency of antigen specific CD4+ T cells. (A) FACS dot plots of negative control corresponding to Fig 2A, including some repeates. (B) FACS dot plots from extended subjects. (C) One example of T regulatory (Treg) cells in tetramer positive CD4+ T cells. PBMCs were stimulated with 6.25 μM RFX4 peptide in a 96-well plate (1-2.5×$10^6$ cells/ml) for 10 days at 37°C, 5% CO2. 20 IU/ml IL2 was supplemented from day 8 to day 10. Cultured cells were incubated with PE-labelled tetramer DQ0602-with or without peptide for 90 minutes at 37°C, 5% CO2, followed by staining with BV421 anti-CD3, FITC anti-CD4 and AF700 anti-CD8 antibodies on ice. Live single CD3+ T cells with percentage of each quadrant population are shown. For Tregs, cells were fixed after tetramer DQ0602 and cell surface marker (BV421 anti-CD3, FITC anti-CD4 and AF700 anti-CD8, PE-CY594 anti-CD127, PE/Cy5 anti-CD25) staining, followed by staining with AF647 anti-FOXP3 and flow cytomerty. All FACS plots were analyzed with FlowJo (version 10.0.8, Becton, Dickinson and Company). (D) Frequency of antigen specific CD4+ T cells from tetramer DQ0602-RFX4-86, RFX4-95 and RFX4-60. Percentage of tetramer+CD4+ in live single CD3+ T cells was analyzed (Dataset 2). Mean value was shown in red and the standard error of the mean (SEM) was shown in error bars. Plots were analyzed with R (version 4.0.3, R Foundation for Statistical Computing, Vienna, Austria., 2020).

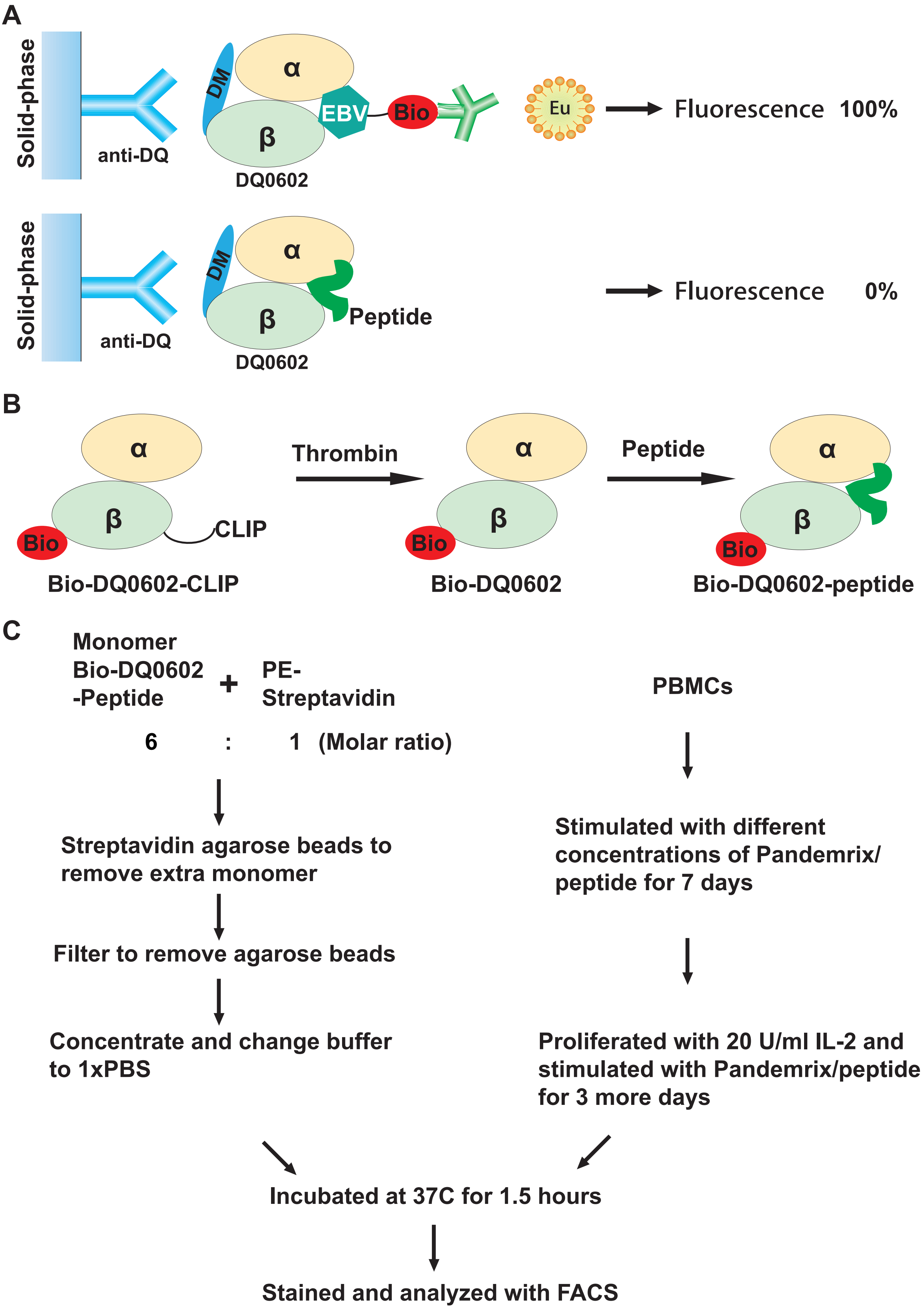
A
Solid-phase
anti-DQ
DM
α
β
EBV
Bio
Eu
DQ0602
Fluorescence 100%
Solid-phase
anti-DQ
DM
α
β
Peptide
DQ0602
Fluorescence 0%
B
α
β
Bio
CLIP
Bio-DQ0602-CLIP
Thrombin
α
β
Bio
Bio-DQ0602
Peptide
α
β
Bio
Bio-DQ0602-peptide
C
Monomer Bio-DQ0602 -Peptide + PE-Streptavidin
6 : 1 (Molar ratio)
Streptavidin agarose beads to remove extra monomer
Filter to remove agarose beads
Concentrate and change buffer to 1xPBS
PBMCs
Stimulated with different concentrations of Pandemrix/ peptide for 7 days
Proliferated with 20 U/ml IL-2 and stimulated with Pandemrix/peptide for 3 more days
Incubated at 37C for 1.5 hours
Stained and analyzed with FACS

**Supplemental Fig. 7**. Diagram of peptide binding assay, peptide exchange, tetramer generation and staining. (A) For competing binding assay, DQ0602 was incubated with biotinylated EBV epitope (Bio-$EBV_{486-500}$, Bio-(GGG)RALLARSHVERTTDE) with (top panel) or without (bottom panel) competing peptide, followed by incubation with monoclonal anti-DQ (SPV-L3) antibody a high binding 96-well plate. DELFIA® time-resolved fluorescence (TRF) intensity was detected after successive incubation with Europium (Eu)-labelled streptavidin and enhancement solution. Plate was washed extensively to remove nonspecific binding. Bio-$EBV_{486-500}$ alone is as the reference (positive control) and without any peptide is as negative control. Compared with the reference, competing peptides with lower than 25% and 25%-50% of fluorescence are considered as strong and weak peptides, respectively. (B) For peptide exchange, biotinylated-DQ0602 (bio-DQ0602) was cleaved with thrombin to remove CLIP, and then incubated with peptide of interest. (C) Tetramer DQ0602-peptide generation afte peptide exchange and PBMCs stimulation and staining with tetramer. Diagram was generated with Illustrator (Version 16.0.0, Adobe).
